# From constitutive killing to microenvironment-activated antimicrobial activity: a critical narrative review of antimicrobial dental materials for caries prevention

**DOI:** 10.3389/fcimb.2026.1983359

**Published:** 2026-10-12

**Authors:** Xiwen Xin, Ziying Tang, Yuejia Deng, Yulong Zhang, Hai Zhang, Bennett T. Amaechi, Andre V. Ritter, Alvin G. Wee, Justin Kaspar, Jing Zhao, Andréa Ferreira Zandoná, Xuelian Huang

**Affiliations:** 1 College of Dentistry, The Ohio State University, Columbus, OH, United States; 2 School of Dentistry, University of Washington, Seattle, WA, United States; 3 Division of Orthodontics, College of Dentistry, The Ohio State University, Columbus, OH, United States; 4 The ADA Forsyth Institute, Somerville, MA, United States; 5 Department of Comprehensive Dentistry, University of Texas at San Antonio School of Dentistry, San Antonio, TX, United States; 6 Department of Restorative Dentistry, School of Dentistry, University of Washington, Seattle, WA, United States; 7 Division of Biosciences, College of Dentistry, The Ohio State University, Columbus, OH, United States; 8 Department of Biomedical Informatics, College of Medicine, The Ohio State University, Columbus, OH, United States; 9 Division of Restorative and Prosthetic Dentistry, College of Dentistry, The Ohio State University, Columbus, OH, United States

**Keywords:** antimicrobial dental materials, caries prevention, cariogenic biofilm, clinical translation, dental caries, nanomaterials, *Streptococcus mutans*, white spot lesions

## Abstract

Dental caries remains one of the most prevalent diseases worldwide. It is a biofilm-mediated, diet-modulated, multifactorial, dynamic disease that poses a substantial public health burden. The ecological plaque hypothesis views caries as a consequence of sugar-induced disruption of oral biofilm homeostasis. Accordingly, plaque control is a key element in caries prevention. Conventional restorative treatment repairs damaged structure but does not directly control the underlying biofilm, motivating development of antimicrobial and antifouling dental materials. This critical narrative review examines seven caries-relevant classes: quaternary ammonium compounds (QACs), including quaternary ammonium polyethyleneimine nanoparticles; metallic nanoparticles; photocatalytic titanium-based materials; graphene-based materials; zwitterionic polymers; biomimetic nanozymes; and antimicrobial peptides. Six material-intrinsic classes are compared using two conceptual axes: predominant antimicrobial action (diffusible, contact-dependent, or antifouling) and activation trigger (constitutive, light-induced, or biofilm-microenvironment-responsive). Antimicrobial peptides are considered separately because their activity is encoded by peptide sequence and molecular design rather than an intrinsic material property. Across classes, *in vitro* antimicrobial, antibiofilm, or antifouling effects are common, but direct evidence of caries prevention is substantially more limited. QACs and metallic nanoparticles have comparatively mature restorative-incorporation evidence; photocatalytic titanium and nanozymes provide triggered catalytic activity; zwitterionic polymers primarily reduce microbial adhesion; graphene remains largely preclinical; and peptide strategies increasingly emphasize pathogen targeting, virulence modulation, and ecological selectivity. Among antimicrobial peptides, C16G2, a specifically targeted antimicrobial peptide directed against *Streptococcus mutans*, has progressed further clinically, whereas other caries-relevant peptides remain predominantly preclinical. Major translational gaps include inconsistent outcome measures, limited independent replication, incomplete formulation-specific therapeutic-window assessment, and reliance on microbiological rather than disease-level endpoints. Antimicrobial or antibiofilm activity should therefore be interpreted as surrogate evidence unless accompanied by reduced enamel demineralization, lesion development or progression, or caries incidence/arrest. Future studies should prioritize standardized testing, matched efficacy and host-safety assessment, independent validation, and clinically meaningful anticaries endpoints.

## Introduction

Dental caries is a multifactorial, diet-modulated, biofilm-mediated disease driven by a persistent imbalance between demineralization and remineralization. Frequent sugar exposure can disrupt biofilm homeostasis and favor a cariogenic community, supporting biofilm management and dietary control as central preventive strategies (Huang and Zandoná, 2025). Dental caries remains the most prevalent condition evaluated by the Global Burden of Disease Study (Centers for Disease Control and Prevention, 2024; GBD 2021 Oral Disorders Collaborators, 2025).

Restorative treatment repairs cavitated tooth structure but does not eliminate the underlying biofilm-mediated disease process. Secondary caries at restoration margins is a major cause of restoration failure, accounting for more than 40% of failures overall and up to 73.9% of composite replacements in long-term studies (Kopperud et al., 2012; Featherstone, 2022). Biofilm-retentive prosthetic and orthodontic appliances create additional caries-prone sites (Preshaw et al., 2011). In orthodontic patients, a meta-analysis of 57 studies involving 9,101 individuals reported white spot lesions in 55% of treated versus 29% of untreated patients, with bonded appliances associated with approximately sevenfold higher odds (Hussain et al., 2025). These settings provide a clinical rationale for materials that actively interfere with cariogenic biofilms rather than functioning only as inert restorative or appliance components.

Accordingly, restorative-material design has progressed from largely inert formulations to antimicrobial and multifunctional bioactive systems that may combine microbial control with remineralization (Melo et al., 2023). Remineralizing agents such as nanohydroxyapatite have shown efficacy in early-lesion models and controlled clinical studies (Amaechi et al., 2019a; Amaechi et al., 2025), but bioactive material design must balance biological activity against esthetic and physicomechanical requirements (Melo et al., 2023).

This narrative review examines seven classes of caries-relevant antimicrobial or antifouling strategies. Six are material-intrinsic: quaternary ammonium compounds (QACs)/quaternary ammonium polyethyleneimine (QPEI) nanoparticles, metallic nanoparticles, photocatalytic titanium-based materials, graphene-based materials, zwitterionic polymers, and biomimetic nanozymes. They are compared by mode of activity (diffusible, contact-dependent, or antifouling) and activation trigger (constitutive, light-activated, or biofilm-microenvironment-responsive).

Antimicrobial peptides constitute the seventh class considered in this review. Unlike the six material-intrinsic classes, their activity is encoded by peptide sequence and molecular design rather than arising from an intrinsic physicochemical property of a dental material. C16G2, a specifically targeted antimicrobial peptide (STAMP) directed against *Streptococcus mutans*, is emphasized because it has progressed further clinically than other caries-relevant antimicrobial peptides. Bioactive glass, smart pH-responsive polymeric delivery systems, organoselenium-based materials, and emerging CRISPR-based targeted antimicrobial platforms are discussed briefly outside the seven-class framework because they either do not represent distinct material-intrinsic antimicrobial chemistries or have a more limited caries-focused evidence base.

The review focuses primarily on *S. mutans*, *Candida albicans*, cariogenic lactobacilli, and caries-relevant multispecies biofilms. *C. albicans* is included because its interaction with *S. mutans* can enhance biofilm cariogenicity and enamel demineralization (Falsetta et al., 2014). Microbiological outcomes—including organism counts, biofilm biomass, metabolic activity, and plaque accumulation—are treated as surrogate evidence unless accompanied by a tooth- or disease-level anticaries endpoint.

This is a narrative, mechanism-driven review rather than a PRISMA-guided systematic review; no protocol was registered and no formal quality-appraisal instrument was applied. Articles were selected for mechanistic relevance and translational value, so statements about evidence gaps refer to the literature included in this synthesis. For cross-class comparison, **Figure 1** maps the six material-intrinsic classes by mode of activity (Ramburrun et al., 2021) and activation trigger (Montoya et al., 2023). The framework is a comparative heuristic, not a mutually exclusive taxonomy: metallic nanoparticles, graphene-based systems, and TiO_2_-containing materials can exhibit overlapping or formulation-dependent mechanisms. Placement therefore reflects the predominant mechanism and activation condition emphasized in the studies reviewed.

**Figure 1:**
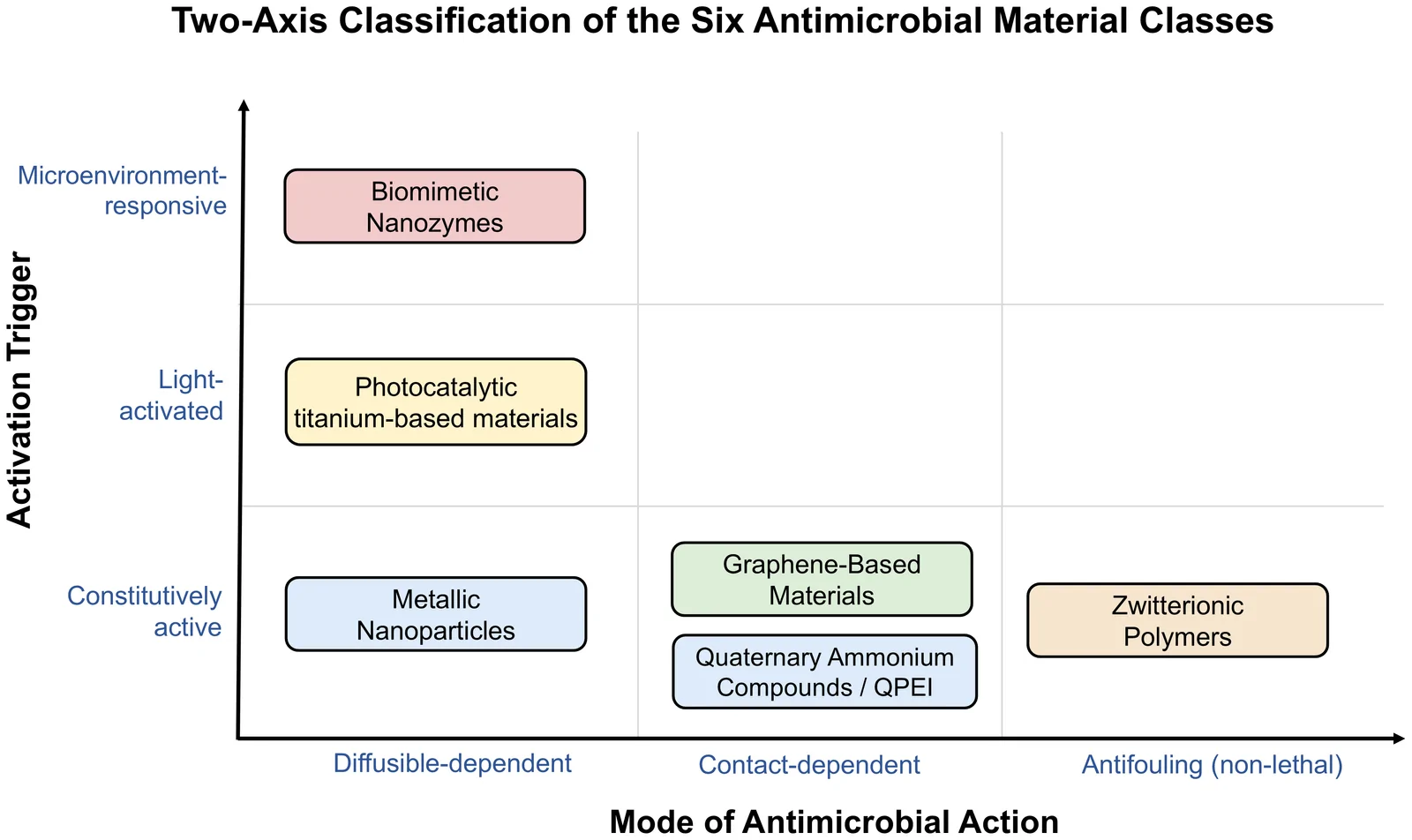
Two-axis classification of the six material-intrinsic antimicrobial classes surveyed in this review, mapped by mode of antimicrobial action (horizontal) and activation trigger (vertical). Titanium-based systems are classified as photocatalytic only when their antimicrobial activity was specifically designed or evaluated as light-induced. Antimicrobial peptides, the seventh class considered here, rely on peptide-encoded targeting and/or antimicrobial activity rather than a material-intrinsic mechanism and therefore fall outside this taxonomy.

The categories are not mutually exclusive; placement reflects the predominant mechanism and activation condition emphasized in the cited studies, and individual formulations may span more than one category.

**Figure 1** highlights that most material classes are constitutively active, whereas photocatalytic titanium-based materials and biomimetic nanozymes depend on external or microenvironmental activation. It also shows that the literature has emphasized microbial killing more than antifouling, with zwitterionic polymers representing the principal non-killing approach.

**Figure 2** distills three recurring interfacial mechanisms: contact-mediated membrane disruption, reactive oxygen species (ROS) generation, and an antifouling hydration barrier. QAC/QPEI systems primarily illustrate contact-mediated killing; photocatalytic titanium-based materials and nanozymes illustrate catalytic ROS generation; and zwitterionic polymers resist initial attachment through surface hydration. Metallic nanoparticles and graphene-based materials can combine more than one of these mechanisms. This mechanistic distinction also informs the antimicrobial-resistance and stewardship considerations discussed later.

**Figure 2:**
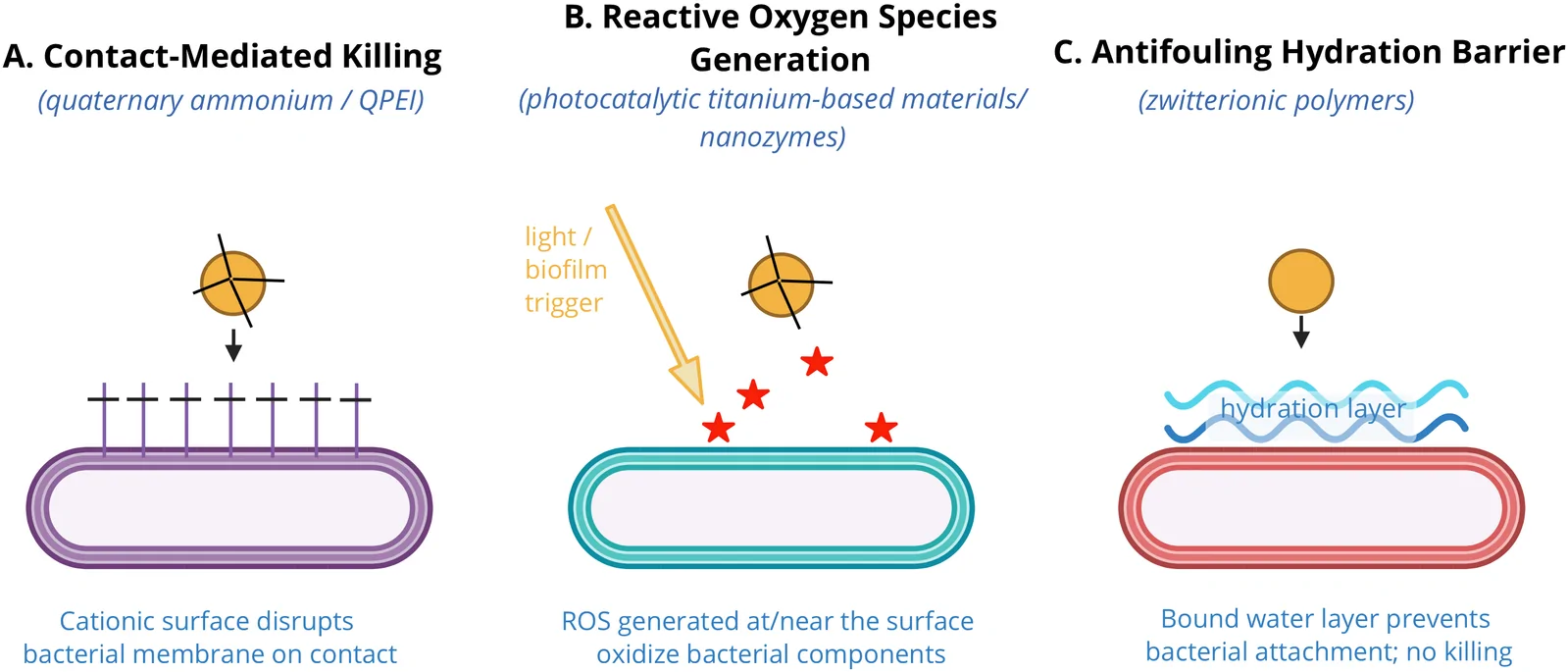
Representative antimicrobial mechanisms of material-intrinsic strategies. **(A)** Contact-mediated killing by quaternary ammonium compounds and QPEI, in which cationic surfaces disrupt bacterial membranes upon contact. **(B)** Reactive oxygen species (ROS) generation by photocatalytic titanium-based materials and nanozymes, in which ROS generated at or near the material surface oxidize bacterial components. Metallic nanoparticles can involve both contact-associated effects **(A)** and ROS generation **(B)**, whereas graphene-based materials primarily resemble the membrane-disruptive mechanism in panel **(A)**, with a secondary oxidative component related to panel **(B)**. **(C)** Antifouling activity of zwitterionic polymers, in which a bound hydration layer inhibits bacterial attachment without directly killing bacteria.

The following sections examine each material class using an integrated mechanism-and-evidence format, with detailed concentrations and quantitative outcomes consolidated in the corresponding tables. Class-specific critical evaluations are incorporated into the Evidence Summary or concluding portion of each material section to interpret evidence quality, safety, translational status, and remaining limitations. Throughout, microbiological surrogate outcomes are distinguished from direct anticaries endpoints such as enamel demineralization, lesion progression, caries arrest, or caries incidence.

## QACs and QPEI nanoparticles

QACs are among the oldest antibacterial chemistries studied in dentistry. They share a permanently cationic nitrogen center that disrupts the bacterial cell envelope through electrostatic interactions rather than through a specific molecular target (Imazato et al., 1994). In caries preventive dental materials, QACs have been incorporated in three main forms: small, diffusible surfactants with limited oral substantivity; polymerizable QAC methacrylate monomers, ranging from methacryloyloxydodecylpyridinium bromide (MDPB) to dimethylaminoalkyl (DMA) methacrylates with varying alkyl chain lengths, that become covalently incorporated into the resin network; and QPEI, in which multiple QAC groups are attached to a cross-linked nanoparticle carrier (Beyth et al., 2006).

The two immobilized subclasses—polymerizable QAC monomers and QPEI nanoparticles—were developed through distinct research lineages (Drejka et al., 2024). Despite their different origins, both rely on the same underlying electrostatic mechanism and are therefore considered together in this review.

### Mechanism and caries-relevant antimicrobial evidence

All QAC subclasses share the same basic mechanism of action: the permanently cationic quaternary nitrogen groups bind to negatively charged phospholipids and teichoic acids in the bacterial envelope, disrupting membrane integrity, promoting cytoplasmic leakage, and ultimately causing cell death (Beyth et al., 2006; Zhang et al., 2016). In the immobilized subclasses, this activity occurs at the material surface without release of the antimicrobial compound. For example, MDPB-containing resin released no detectable MDPB after curing yet retained contact-dependent activity against *S. mutans* (Imazato et al., 1994; Imazato, 2003).

Across polymerizable QAC homologues, antibacterial activity generally increased with alkyl-chain length up to CL16 and declined at CL18, a pattern attributed to greater membrane interaction followed by partial shielding of cationic groups in the longest chains (Tiller et al., 2001; Li et al., 2013; Zhang et al., 2016). DMAHDM (CL16) was therefore the most active homologue tested. QPEI achieves high cationic charge density through multiple quaternary ammonium groups on a cross-linked nanoparticle, with efficacy influenced by particle distribution and surface accessibility; controlled neutralization and surface treatment can reduce the loading required for activity (Beyth et al., 2006; Shvero et al., 2015; Zaltsman et al., 2016).

Despite these differences in molecular architecture, both subclasses share the same fundamental design constraint: antimicrobial activity depends on presenting enough accessible, unshielded cationic groups at the material surface.

#### Monomeric quaternary ammonium methacrylates

Imazato’s foundational studies established that MDPB exhibits strong contact-dependent activity against *S. mutans*, with no detectable leaching and no adverse effect on bond strength (Imazato et al., 1994; Imazato, 2003). Longer-chain QAC monomers generally showed greater antibacterial activity, and DMAHDM-containing remineralizing composites produced substantial reductions in viable bacteria and metabolic activity without compromising the measured mechanical properties (Zhang et al., 2016). Concentrations and quantitative outcomes are summarized in **Table 1**. These findings demonstrate antibacterial activity, but microbiological reductions should not by themselves be interpreted as evidence that the formulation prevents enamel demineralization or clinical caries.

**Table 1:** Antibacterial, antifungal, and cytotoxicity evidence for monomeric quaternary ammonium compounds in caries-relevant applications.

Material/system	Application	Target organism(s)	Effective conc.	Cytotoxic conc. tested	Key result	Ref.
MDPB (methacryloyloxydodecylpyridinium bromide), copolymerized	Dentin primer/adhesive resin	*S. mutans*	5% MDPB (in primer)	No cytotoxicity data were identified for this formulation; CTAB/DODAB cytotoxicity data are not formulation-matched to MDPB.	Complete kill after 30 s contact with 5% MDPB-incorporated primer; no leaching detected after curing; bond strength preserved or improved at 1–2%	(Imazato et al., 1994; Imazato, 2003)
quaternary ammonium methacrylate series, chain lengths 3–18 (DMAPM, DMAHM, DMADDM, DMAHDM, DMAODM)	Experimental bonding agent	Dental plaque microcosm biofilm	Not specified (chain-length comparison; concentration not reported)	N.A.	Antibacterial potency increased with chain length up to CL16 (DMAHDM), then declined at CL18	(Li et al., 2013)
DMAHDM (CL16), 3 wt% with 20% NACP	Calcium-phosphate remineralizing nanocomposite	Total microorganisms, total streptococci, mutans streptococci	3 wt% DMAHDM (with 20% NACP)	Not directly tested; low leaching for DMAHDM has been inferred from covalent copolymerization rather than demonstrated by concentration-matched cytotoxicity testing.	2-log reduction in biofilm CFU; 10-fold reduction in metabolic activity and lactic acid production; flexural strength and elastic modulus matched commercial control	(Zhang et al., 2016)
DMAHDM-containing composite, 20 passages	Resistance-induction assay	*S. mutans*, *S. sanguinis*, *S. gordonii*	Not specified (resistance-induction protocol; concentration not reported)	Not directly tested; available DMAHDM safety discussion is inferential and does not provide a concentration-matched cytotoxicity value for this resistance-induction protocol.	No drug resistance induced after 20 passages, in contrast to chlorhexidine, which induced resistance in multiple species under the same protocol	(Wang S. et al., 2018)
DMADDM, 5–20 wt%	Heat-polymerized denture base resin	*C. albicans* biofilm	5–20 wt% DMADDM	N.A.	Significant reduction in CFU and XTT metabolic activity; reduced hyphal formation by SEM; flexural strength, bond quality, and surface roughness unaffected	(Chen H. et al., 2017)
DMADDM	pH-dependent antifungal mechanism study	*C. albicans* (planktonic and murine oropharyngeal candidiasis model)	Not specified (pH-dependent; specific test concentration not reported in text)	N.A.	Antifungal potency markedly pH-dependent; suppressed oropharyngeal candidiasis *in vivo*, though less potent against *C. albicans* than against the bacterial species tested at comparable concentrations	(Chen et al., 2020b)
CTAB-modified resin composite eluates	Cytotoxicity/genotoxicity screening	Human cell lines (not bacterial)	N/A (cytotoxicity/genotoxicity study, not an antibacterial efficacy test)	Up to 2 wt% (eluate assay; dose-dependent cytotoxicity/genotoxicity)	Dose-dependent cytotoxicity, apoptosis/necrosis induction, and pronounced genotoxicity	(Szymczak-Pajor et al., 2025)
DODAB-modified resin composite eluates	Cytotoxicity/genotoxicity screening	Human cell lines (not bacterial)	N/A (cytotoxicity/genotoxicity study, not an antibacterial efficacy test)	Up to 2 wt% (eluate assay; minimal, non-significant effect)	Minimal, statistically non-significant cytotoxicity, apoptosis, or genotoxicity up to 2 wt%	(Szymczak-Pajor et al., 2025)

Outcomes summarized as antibacterial/antifungal effects are microbiological surrogates unless a caries-specific endpoint is explicitly reported.

In a serial-passage study, repeated exposure to a DMAHDM-containing composite did not produce a detectable increase in antimicrobial resistance in *S. mutans* under the conditions tested, whereas chlorhexidine selected for reduced susceptibility in multiple oral bacterial species (Wang S. et al., 2018). Comparable resistance-selection data were not identified for QPEI. Evidence regarding species selectivity also remains limited: DMAHDM altered the relative abundance of *S. mutans* within a biofilm community, but it remains unclear whether this reflected differential susceptibility or changes in interspecies competition after overall biomass reduction (Wang et al., 2019). The corresponding formulation and quantitative findings are provided in **Table 1**.

#### QPEI nanoparticles

Beyth and colleagues demonstrated that QPEI-containing resin composites exert strong antibacterial activity against *S. mutans* without compromising the measured flexural properties (Beyth et al., 2006). Subsequent studies reported favorable surface properties and biocompatibility (Yudovin-Farber et al., 2008), lower-loading synthetic variants (Zaltsman et al., 2016), sustained activity over time (Yudovin-Farber et al., 2010; Shvero et al., 2015), and reproducibility across composite systems (Pietrokovski et al., 2016; Chladek et al., 2023). A human *in situ* study also demonstrated reduced biofilm vitality (Beyth et al., 2010). Detailed concentrations and quantitative outcomes are summarized in **Table 2**. However, because QPEI has not been evaluated in randomized controlled trials assessing caries incidence or restoration survival, its clinical evidence remains limited. The *in situ* biofilm response is a surrogate microbiological endpoint and does not establish anticaries efficacy in the absence of a demineralization or caries outcome.

**Table 2:** Antibacterial and antifungal evidence for QPEI nanoparticles and structurally related quaternary ammonium systems in caries-relevant applications.

Material/system	Application	Target organism(s)	Effective conc.	Cytotoxic conc. tested	Key result	Ref.
Classic octylated/quaternized QPEI, 1 wt%	Resin composite	*S. mutans*	1 wt% QPEI	Not specified; related work from the same research lineage reports favorable biocompatibility, but no formulation-matched cytotoxicity concentration was provided (Yudovin-Farber et al., 2008).	Strong antibacterial activity; flexural modulus and flexural strength preserved in tested commercial materials	(Beyth et al., 2006)
QPEI synthesis variants (neutralized, N-lauroylsarcosine-treated)	Acrylate- and epoxy-based dental materials	General oral bacteria	As low as 0.5 wt%	N.A.	Antibacterial efficacy achieved at loadings as low as 0.5 wt%, roughly half the original formulation’s requirement	(Zaltsman et al., 2016)
QPEI, 0.5–2 wt%	Unfilled Bis-GMA/TEGDMA matrix	N/A (physicochemical study)	N/A (physicochemical/mechanical study; no antibacterial testing reported)	N.A.	Degree of conversion essentially unaffected; solubility increased; flexural strength and impact resistance reduced as loading rose	(Barszczewska-Rybarek et al., 2021)
QPEI, 0.5–3 wt%	Glass-filled experimental dimethacrylate composite	General oral bacteria	0.5–3 wt% QPEI	0.5–3 wt% (same study); ‘satisfactory biocompatibility’ reported even at loadings up to 3 wt% that reduced flexural/compressive strength	Complete reduction in bacterial adherence and satisfactory biocompatibility; water sorption/solubility increased and flexural/compressive strength reduced at highest loading	(Pietrokovski et al., 2016)
QPEI, *in situ* intraoral exposure	Composite specimens worn intraorally (human)	Oral biofilm (mixed flora)	1 wt% QPEI (per corresponding narrative)	Same *in situ* study (1 wt% QPEI); no adverse impact noted, but this measured biofilm vitality/exposure outcome rather than a dedicated cytotoxicity assay	Biofilm vitality reduced by more than 50% after short-term intraoral exposure	(Beyth et al., 2010)
QPEI, 1 wt%	Glass ionomer cement	*S. mutans*, *L. casei*	1 wt% QPEI	N.A.	Long-lasting activity for at least 1 month; no inhibition halo, consistent with non-diffusible mechanism	(Beyth et al., 2012)
QPEI nanoparticles	Resin composite, particle-distribution study	General oral pathogens	Not specified	N.A.	Antibacterial effect detected at both outer and inner surfaces; XPS confirmed particle distribution through bulk material, not only exposed surface	(Shvero et al., 2015)
QPEI nanoparticles	Composite foundation material	General oral bacteria	Not specified	N.A.	Antibacterial effect confirmed in a foundation-material application distinct from bulk restorative composite	(Pietrokovski et al., 2016)
Polyethyleneimine nanoparticles	Temporary restorative materials	General oral bacteria	Not specified	N.A.	Improved antibacterial performance relative to unmodified temporary restorative materials	(Abramovitz et al., 2013)
QPEI, low-level addition	Endodontic sealers	General endodontic pathogens	Not specified (described only as ‘low-level addition’ in source studies)	N.A.	Antibacterial and antibiofilm activity, with formulation-specific mechanical trade-offs varying by sealer base chemistry	(Kesler Shvero et al., 2013; Barros et al., 2014b; Barros et al., 2014a)
QPEI, 1 wt%	Orthodontic cement adjacent to brackets	*S. mutans*, *L. casei*	1 wt% QPEI	N.A.	Significant reduction in bacterial growth adjacent to brackets in an *ex vivo* tooth-bracket model	(Sharon et al., 2018)
Quaternized PEI (QPEI-C6), non-dental chemistry	General antimicrobial mechanism study	*C. albicans*	16 μg/mL (MIC)	N.A.	MIC of 16 μg/mL, within the same order of magnitude as its bactericidal MICs (8–128 μg/mL)	(Gao et al., 2007)
C12BDMA-Cl (structurally related quaternary ammonium compound), 3–5 wt%	Denture base resin	*C. albicans*	3 wt%	N.A.	Antifungal activity at 3 wt% without mechanical deterioration	(Aldulaijan et al., 2024)
QA10+TMXDI/QA12+TMXDI urethane-dimethacrylate copolymers	Covalently copolymerized restorative comonomer	*S. aureus*, *E. coli*	Not specified (comonomer loading/ratio not given in wt% terms in text)	N.A.	QA10+TMXDI showed the greatest antibacterial activity among the tested variants; water sorption and leachability increased, but the measured physicochemical and mechanical properties remained within the reported acceptable ranges.	(Drejka et al., 2024)

The two *C. albicans* rows (quaternized PEI, non-dental chemistry; C12BDMA-Cl, a structurally related but distinct compound) are indirect, hypothesis-supporting evidence rather than direct data for the dental QPEI nanoparticle platform itself, which has no *C. albicans* data of its own; see text. These antimicrobial outcomes should not be interpreted as direct evidence of anticaries efficacy. MIC, minimum inhibitory concentration.

QPEI performance is formulation-dependent: higher loading can increase solubility and compromise mechanical properties (Pietrokovski et al., 2016; Barszczewska-Rybarek et al., 2021). Applications include glass ionomer cements, temporary restoratives, endodontic sealers, and orthodontic models (Beyth et al., 2012; Abramovitz et al., 2013; Kesler Shvero et al., 2013; Barros et al., 2014b; Barros et al., 2014a; Sharon et al., 2018). Because *C. albicans* can interact synergistically with *S. mutans*, antifungal findings from related quaternary ammonium chemistries are also relevant (Gao et al., 2007; Falsetta et al., 2014; Chen H. et al., 2017; Chen et al., 2020b; Aldulaijan et al., 2024). Formulation-specific concentrations and outcomes are summarized in **Tables 1** and **2**.

### Evidence summary

The antibacterial, antifungal, and cytotoxicity evidence for monomeric quaternary ammonium methacrylates is summarized in **Table 1**, while **Table 2** presents the corresponding evidence for QPEI nanoparticles and closely related quaternary ammonium chemistries.

The absence of bacterial resistance development with DMAHDM, in contrast to chlorhexidine, represents a separate safety-relevant consideration (Wang S. et al., 2018). Thus, the two immobilized subclasses should not be regarded as interchangeable. Their distinct toxicity profiles and evidence bases warrant separate evaluation. Antifungal findings from structurally related quaternized PEI systems should not be interpreted as direct evidence for the dental QPEI nanoparticle platform.

Overall, QAC/QPEI systems have a comparatively mature restorative-incorporation literature, including human *in situ* evidence for QPEI, but most outcomes remain microbiological rather than caries-specific (Beyth et al., 2010). Their main translational constraints are formulation-dependent efficacy, mechanical trade-offs at higher loading, and an incompletely defined therapeutic window; toxicity data from CTAB or DODAB should not be generalized to polymerizable DMAHDM/DMADDM or dental QPEI formulations (Yudovin-Farber et al., 2008; Yudovin-Farber et al., 2010; Pietrokovski et al., 2016; Zhang et al., 2016; Chen H. et al., 2017; Wang S. et al., 2018; Chen et al., 2020b; Chladek et al., 2023; Szymczak-Pajor et al., 2025). Contact-dependent activity may also be influenced by salivary pellicle, although QPEI retained substantial antibiofilm activity during whole-saliva exposure (Beyth et al., 2010; Li et al., 2014).

## Metallic nanoparticles

Metallic and metal oxide nanoparticles are among the most extensively studied antimicrobial additives for controlling cariogenic biofilms. Their high surface-area-to-volume ratio, surface reactivity, and, for some formulations, ion-release properties contribute to antimicrobial activity in dental adhesives, composites, and cements (Chen et al., 2020a). This section focuses primarily on elemental silver nanoparticles (AgNPs) and metal oxide nanoparticles such as zinc oxide (ZnO), magnesium oxide (MgO), and copper oxide (CuO), whose reported antimicrobial activity is constitutive and does not require deliberate light activation. Titanium-based systems whose primary antimicrobial mechanism depends on photocatalytic activation are considered separately in the following section. A non-photocatalytic TiO_2_ formulation is included here only because the cited study evaluated antibacterial activity without attributing the observed effect to light-induced photocatalysis.

### Mechanisms and caries-relevant antimicrobial evidence

Silver nanoparticles act through at least two mechanisms though these have not always been experimentally distinguished. Direct interaction with the bacterial surface increases membrane permeability and can lead to cell death, whereas Ag^+^ ions released from the particles can independently interfere with metabolic and enzymatic processes (Corrêa et al., 2015). Zinc oxide nanoparticles add oxidative stress to a similar contact-mediated mechanism. In *S. mutans* biofilms, ZnO nanoparticles increase intracellular ROS while disrupting membrane potential, energy metabolism, and extracellular polysaccharide synthesis, indicating activity against multiple cellular processes rather than a single molecular target (Emram et al., 2025).

TiO_2_ nanoparticles, discussed separately below as a photocatalytic material class, also exhibit baseline antibacterial activity through surface-associated oxidative stress, even in the absence of strong light activation (Sodagar et al., 2017). Magnesium oxide and copper oxide nanoparticles appear to act through a combination of alkalinity modulation, ion release, and oxidative stress, although much of the available mechanistic evidence is derived from narrative reviews rather than dedicated mechanistic studies (Ma et al., 2022; Sharifian et al., 2023). Nanoparticles containing zinc or magnesium have also been proposed to modify the local pH and thereby reduce demineralization. This represents an indirect anticaries effect that is distinct from direct bacterial killing (Chen et al., 2020a).

When incorporated into adhesives, composites, cements, sealants, and topical formulations, metallic and metal oxide nanoparticles have reduced bacterial adhesion or biofilm formation relative to unmodified controls in multiple *in vitro* studies (Bolenwar et al., 2023). Silver and zinc oxide nanoparticles account for much of the restorative-incorporation evidence, including flowable composites, self-etch adhesives, and glass ionomer cements (Kasraei et al., 2014; Porter et al., 2020; Wang et al., 2022; Wassel and Allam, 2022). TiO_2_ nanoparticles have also been evaluated in an orthodontic composite without specified photoactivation and are included here because the reported antibacterial effect was not attributed to photocatalysis (Sodagar et al., 2017). Formulation-specific concentrations and quantitative outcomes are summarized in **Table 3**, allowing the text to focus on the overall evidence pattern and its translational interpretation.

**Table 3:** Antibacterial evidence for metallic nanoparticles in caries-relevant applications.

Material/system	Application	Target organism(s)	Effective conc.	Cytotoxic conc. tested	Key antibacterial result	Ref.
Silver nanoparticles (AgNPs)	General antibacterial mechanism	Cariogenic bacteria (general)	Not specified	0.05% AgNP in primers/adhesives — no measurable effect on human gingival fibroblast viability (Corrêa et al., 2015) (same source reference)	Membrane disruption via close contact; Ag^+^ release impairs metabolic/enzymatic pathways	(Corrêa et al., 2015)
Zinc oxide nanoparticles (ZnO NPs)	Antibacterial mechanism study	*S. mutans* biofilms	Not specified	Not specified in source; dose- and time-dependent cytotoxicity reported, with ZnO showing the lowest viability among TiO_2_/SiO_2_/ZnO/Al_2_O_3_ tested on human dental pulp stem cells (Tabari et al., 2017) (different study/cell type)	Increased intracellular ROS; disrupted membrane potential; reduced ATP; downregulated EPS synthesis	(Emram et al., 2025)
AgNP and ZnO NP, 1% each	Flowable composite resin	*S. mutans*, *Lactobacillus*	1% AgNP and 1% ZnO NP	No cytotoxicity data were identified for this 1% AgNP/ZnO composite formulation; available data for 0.05% AgNP in primers/adhesives and for ZnO in dental pulp stem cells involve different formulations (Corrêa et al., 2015; Tabari et al., 2017).	Significant antibacterial activity by direct contact test at 1% loading	(Kasraei et al., 2014)
AgNP, 0.05% and 0.1%	Universal self-etch adhesive	*S. mutans*	0.05% and 0.1%	0.05% AgNP in primers/adhesives — no measurable effect on human gingival fibroblast viability (Corrêa et al., 2015); 0.1% not tested for cytotoxicity	Antibacterial activity with preserved resin-dentin bond strength	(Wang et al., 2022)
TiO_2_ nanoparticles (photoactivation not specified)	Orthodontic composite; evaluated without specified photoactivation	*S. mutans*	Not specified	TiO_2_ included in a four-oxide (TiO_2_, SiO_2_, ZnO, Al_2_O_3_) dental pulp stem cell cytotoxicity study showing dose- and time-dependent cytotoxicity; different formulation (nanoparticle suspension vs. orthodontic composite), concentration not specified (Tabari et al., 2017)	Reduced *S. mutans* growth relative to the unmodified composite; the study did not attribute the effect to light-induced photocatalysis, and shear bond strength was evaluated concurrently	(Sodagar et al., 2017)
Nano silver fluoride (NSF)	Topical caries-arrest agent (clinical)	Active carious dentin lesions	Not specified (clinical topical application)	N.A.	72.91% vs. 34% (control) arrested lesions at 5 months; 65.21% vs. 28.88% at 12 months	(Nagireddy et al., 2019)
Nano silver fluoride (NSF) vs. silver diamine fluoride (SDF)	Topical caries-arrest agent (clinical)	Active carious lesions, early childhood caries	Not specified (clinical topical application)	N.A.	Comparable arrest efficacy to SDF with reduced tooth discoloration	(Quritum et al., 2024)
AgNP-incorporated dental varnish, 25 μg/mL	Antifungal mechanism study	*C. albicans*	25 μg/mL	No cytotoxicity data were identified for this varnish formulation; available 0.05% AgNP data involve primers/adhesives and a different concentration unit (Corrêa et al., 2015).	79% membrane permeability; 22.2% ROS production; CFU below 10³ within 5 h; 62.5% biofilm inhibition/degradation	(Subramaniam et al., 2024)
ZnO nanowires, 500–1000 ppm	PMMA denture-base matrix	*C. albicans* (2 strains)	500–1000 ppm	No cytotoxicity data identified for this ZnO nanowire/PMMA formulation (source study assessed antifungal activity and ultrastructure only); a separate ZnO nanoparticle study (different formulation) found lowest viability among 4 oxides tested on dental pulp stem cells (Tabari et al., 2017)	94% biofilm inhibition at 1000 ppm; 80% at 500 ppm; TEM confirms cell-wall/membrane damage	(Apip et al., 2021)
Ag + ZnO + chitosan nanoparticles	Denture tissue conditioner	Bacteria and *C. albicans*	Not specified	No cytotoxicity data were identified for this combined Ag+ZnO+chitosan formulation; available data address AgNP alone and ZnO alone rather than the combined tissue-conditioner formulation (Corrêa et al., 2015; Tabari et al., 2017).	Inhibited both; fungal growth suppressed more effectively than bacterial growth	(Mousavi et al., 2020)

Antibacterial endpoints are distinguished from direct anticaries outcomes such as caries arrest or enamel demineralization.

Topical applications have substantially more mature clinical evidence than nanoparticle-containing restorative materials. Nano silver fluoride (NSF), which combines silver nanoparticles with fluoride, was more effective than control in arresting pediatric caries at both 5- and 12-month follow-up in randomized trials (Nagireddy et al., 2019). NSF also showed caries-arrest efficacy comparable to silver diamine fluoride (SDF), with less tooth discoloration (Quritum et al., 2024). In contrast, metallic nanoparticles incorporated into restorative materials have not reached a comparable level of clinical validation. A systematic review of antimicrobial restorative materials found consistent evidence of laboratory antimicrobial activity but limited evidence that these effects translate into reduced caries clinically (do Amaral et al., 2016). Thus, within the evidence considered here, randomized clinical trials evaluating a direct caries-arrest endpoint were available for nano silver fluoride (Nagireddy et al., 2019; Quritum et al., 2024). However, these findings apply to a topical formulation and should not be extrapolated to metallic nanoparticles incorporated into restorative materials. This distinction is important: the restorative-material studies summarized above primarily demonstrate antimicrobial activity, whereas the NSF trials measured caries arrest and therefore provide direct anticaries evidence (Nagireddy et al., 2019; Quritum et al., 2024).

The antimicrobial activity of metallic nanoparticles also extends to *C. albicans*. Silver- and zinc oxide-based dental formulations have shown substantial inhibition or disruption of fungal biofilms, and combined silver-zinc oxide-chitosan systems have demonstrated activity against both bacterial and fungal components (Mousavi et al., 2020; Apip et al., 2021; Subramaniam et al., 2024). The quantitative effects are summarized in **Table 3**. These findings indicate that antibacterial and antifungal effects do not necessarily parallel one another within the same formulation. However, these studies did not evaluate caries-specific clinical outcomes; reductions in fungal or bacterial biofilm therefore remain microbiological outcomes and should not be interpreted as evidence of caries prevention without a tooth-level demineralization or caries endpoint.

### Evidence summary

The caries-relevant antibacterial evidence for metallic nanoparticles discussed above is summarized in **Table 3**.

Metallic nanoparticles combine broad antimicrobial activity with extensive formulation flexibility, but clinical maturity is uneven. Topical nano silver fluoride has reached randomized trials with direct caries-arrest endpoints (Nagireddy et al., 2019; Quritum et al., 2024), whereas restorative-incorporated AgNP and ZnO systems remain largely preclinical. Biocompatibility is strongly dependent on particle size, concentration, surface chemistry, ion release, and matrix composition, and current efficacy and toxicity datasets are generally not concentration-matched; therefore, a formulation-specific therapeutic window cannot yet be defined reliably (Corrêa et al., 2015; Tabari et al., 2017; Emram et al., 2025).

## Photocatalytic titanium-based materials

This section addresses titanium-based materials designed or evaluated for light-induced photocatalytic antimicrobial activity, distinguishing them from TiO_2_ formulations tested without specified photoactivation (Sodagar et al., 2017). With appropriate irradiation, photocatalysts generate ROS catalytically rather than by releasing a finite antimicrobial payload (Mohammadi et al., 2025; Qi et al., 2025).

### Photocatalytic mechanism and caries-relevant antimicrobial evidence

Photoexcited TiO_2_ generates electron-hole pairs that produce hydroxyl radicals, superoxide, and related ROS, causing oxidative damage to bacterial membranes, proteins, and nucleic acids. Because this is a multi-target oxidative mechanism rather than a single molecular interaction, conventional target-specific resistance may be less readily selected.

Unmodified TiO_2_ is limited by ultraviolet-dependent activation and rapid electron-hole recombination. Nitrogen doping can extend absorption into the visible range, whereas metal dopants such as silver, copper, bismuth, strontium, or gold can improve charge separation; some metal-containing systems may also add ion-mediated antimicrobial effects (Asahi et al., 2001).

Doped TiO_2_ has shown visible-light antimicrobial activity, and a systematic review of TiO_2_-coated orthodontic brackets found pooled effects against *S. mutans*, *L. acidophilus*, and *C. albicans* despite substantial heterogeneity (Gupta et al., 2013; Solanki et al., 2023). Antifungal activity against *C. albicans* has also been reported in denture-related and 3D-printed platforms (Abdulrazzaq Naji et al., 2018; Moradpoor et al., 2021; Ahmed et al., 2023; Altarazi et al., 2024).

The effectiveness of ROS-generating materials may be limited by the extracellular polymeric substance (EPS) matrix of mature biofilms. EPS components can react with short-lived hydroxyl radicals and singlet oxygen, potentially quenching these species before they reach bacterial cells (da Cruz Nizer et al., 2024). Accordingly, activity measured against planktonic bacteria or relatively thin biofilms may overestimate the activity achievable against mature cariogenic biofilms.

Doped TiO_2_ dental resins and adhesives have shown activity against *S. mutans* under irradiated and, in some formulations, non-irradiated conditions; dark activity was attributed primarily to inherent nanoparticle toxicity rather than photocatalysis (Poosti et al., 2013; Zane et al., 2016; Esteban Florez et al., 2018; Zhao et al., 2023). Functionalization can also support durable filler-matrix interfaces without compromising the measured sorption, solubility, or mechanical properties (Esteban Florez et al., 2020b; Esteban Florez et al., 2020a; Hiers et al., 2022). Quantitative outcomes are summarized in **Table 4**.

**Table 4:** Antibacterial evidence for titanium-based photocatalytic materials in caries-relevant applications, including antifungal evidence against *C. albicans*.

Material/doping system	Application	Target organism(s)	Effective conc.	Cytotoxic conc. tested	Key antibacterial result	Ref.
N-doped TiO_2_ (N-TiO_2_), 50–80% v/v	Dental adhesive resin (OptiBond Solo Plus)	*S. mutans* biofilms	50–80% v/v N-TiO_2_ in adhesive resin	Clinically relevant loading (conc. not specified); no elevated cytotoxic signal found in functionalized nanofilled adhesive (Esteban Florez et al., 2020a)	Superior antibacterial activity at 3 and 24 h, light and dark; functionalized interface preserves sorption and mechanical properties	(Esteban Florez et al., 2018; Esteban Florez et al., 2020b; Esteban Florez et al., 2020a; Hiers et al., 2022)
Sr-N co-doped TiO_2_ + nano-hydroxyapatite filler	Dental resin composite	*S. mutans*	Not specified	N.A.	98.96% antibacterial rate; concurrent remineralization in simulated body fluid	(Zhao et al., 2023)
N-doped TiO_2_ (biocompatibility study)	Dental resin formulation	General oral bacteria	Not specified	Same study reports biocompatibility in “tested formulations”; specific concentration not stated	Biocompatible and antibacterial in dental resin	(Zane et al., 2016)
TiO_2_ nanoparticles, 1%	Orthodontic adhesive (Transbond XT)	*S. mutans*	1% TiO_2_ nanoparticles in orthodontic adhesive	N.A.	Antibacterial with no significant shear-bond-strength penalty	(Poosti et al., 2013)
N-doped TiO_2_ coating	Stainless-steel orthodontic brackets	*S. mutans*	Not specified	Not reported for this specific coating; pooled *in vitro* literature on TiO_2_-coated brackets generally found lower cytotoxicity than uncoated brackets (Solanki et al., 2023) (different study)	Split-mouth RCT*: significantly lower plaque *S. mutans* at 30/60 days (P = .005); 90-day *in vitro* activity sustained	(Salehi et al., 2018; Monica and Padmanabhan, 2022)
Ag co-doped TiO_2_ coating	Stainless-steel orthodontic brackets	*S. mutans*, *P. gingivalis*	Not specified	N.A.	Reduced bacterial adhesion and biofilm formation *in vitro*	(Fatani et al., 2017)
Gold/Pd/Pt-loaded monosodium titanate	Foundational antibacterial panel	Five oral bacterial species	2 μM Pt(IV)-titanate (most potent); growth suppression 15–100% across metal/species pairings	Native titanate: little effect on THP1 monocytes/L929 fibroblasts, but 30–40% metabolic suppression in OSC2 and diploid fibroblast lines (conc. not specified) (Chen et al., 2015) (different study than (Chung et al., 2011))	Native titanate inert; metal-loaded titanate suppressed growth 15–100%, up to ~375-fold more potent than free metal ion	(Chung et al., 2011)
Nanosized gold-titanate (nMST-Au(III))	Cariogenic-bacteria-targeted antibacterial	*S. mutans*, *L. casei*	Not specified (dose-dependent; no single effective concentration stated)	Gold-titanate: added metal-specific cytotoxic suppression beyond native-titanate baseline; no detectable free Au(III) release over 21 days (conc. not specified) (Chen et al., 2015) (different study than (Eiampongpaiboon et al., 2015))	Dose-dependent activity; TEM shows gold-nanoparticle internalization and cell-wall disruption	(Eiampongpaiboon et al., 2015)
Au(III)-titanate, 25 mg/L	Gene-expression/resistance study	*S. mutans*, *L. casei*	25 mg/L	*S. mutans* tolerated up to 200 mg/L before growth arrest; *L. casei* could not adapt to escalating concentrations (Theda et al., 2018) (same study — resistance/tolerance data, not a dedicated mammalian cytotoxicity assay)	Downregulates *L. casei* adhesion genes; *S. mutans* compensates via Pac upregulation and greater tolerance	(Theda et al., 2018)
Nanoparticulate gold-loaded titanate (nMT), 10 wt%	Adhesive primer/bonding resin — material compatibility	N/A (mechanical endpoint)	10 wt% (mechanical/compatibility endpoint, not an antibacterial effect)	N.A.	No effect on shear bond strength (21.9–23.9 MPa) or microleakage	(Deng et al., 2016)
TiO_2_ nanoparticles (green-synthesized)	General oral antifungal	*C. albicans*	Not specified (optimized synthesis conditions; no concentration stated)	N.A.	~73% growth inhibition under optimized synthesis conditions	(Moradpoor et al., 2021)
TiO_2_ nanoparticles, up to 2 wt%	Soft denture liner (PEMA)	*C. albicans*	2 wt%	N.A.	Viable counts fell from 139.8 (control) to 1.0 at 2 wt%; dose-dependent antifungal effect	(Ahmed et al., 2023)
TiO_2_ nanoparticles, 0.10–0.75 wt%	3D-printed denture base resin	*C. albicans*	0.10–0.50 wt% (effective range; effect lost at 0.75 wt%)	N.A.	Significant reduction in surface adhesion at 0.10–0.50 wt%; effect lost at 0.75 wt%	(Altarazi et al., 2024)

*RCT, randomized controlled trial.

The principal unresolved issues are translational: durability of dopants or metal loading, formulation-specific biocompatibility, and whether clinically relevant sites receive sufficient activating light.

Gold- or platinum-loaded monosodium titanates have also suppressed bacterial growth, with nanoscale formulations outperforming micron-scale counterparts and evidence of species-dependent susceptibility (Chung et al., 2011; Eiampongpaiboon et al., 2015; Theda et al., 2018). Tested adhesive formulations did not significantly impair bond strength or microleakage (Deng et al., 2016); formulation details are provided in **Table 4**.

### Orthodontic appliances: clinical-trial evidence

Photocatalytic TiO_2_ has progressed furthest clinically in orthodontics. A split-mouth trial of nitrogen-doped TiO_2_-coated brackets reduced plaque-associated *S. mutans* at 30 and 60 days, although the effect declined thereafter; a 90-day *in vitro* study reported more sustained activity (Salehi et al., 2018; Monica and Padmanabhan, 2022). Importantly, the clinical outcome was microbiological and did not establish prevention of white spot lesions, demineralization, or caries (Monica and Padmanabhan, 2022).

Silver-co-doped TiO_2_ coatings also reduced *S. mutans* and *Porphyromonas gingivalis* adhesion and biofilm formation *in vitro* (Fatani et al., 2017). TiO_2_-coated nickel–titanium archwires showed significantly less *S. mutans* adhesion than uncoated wires after one month of intraoral exposure; however, substantial delamination of the coating occurred during the same period (Venkatesan et al., 2020).

### Intraoral light access

Light availability is a central translational constraint. Exposure is relatively favorable on accessible buccal, labial, and occlusal surfaces and around buccally bonded orthodontic appliances, which are also the sites represented in the available clinical evidence (Fatani et al., 2017; Salehi et al., 2018; Venkatesan et al., 2020; Monica and Padmanabhan, 2022).

Removable prostheses can be irradiated extraorally under controlled conditions, consistent with the concentration of antifungal evidence in denture substrates (Abdulrazzaq Naji et al., 2018; Moradpoor et al., 2021; Ahmed et al., 2023; Altarazi et al., 2024). By contrast, proximal, subgingival, deep fissure, cervical, and subsurface restorative sites may receive little light, creating a mismatch between photocatalytic activation and several caries-prone locations.

Intraoral irradiance at these candidate sites has not been directly quantified in the studies reviewed. Interpretation is further complicated because violet-blue light itself can suppress *S. mutans* biofilms and reduce enamel or dentin demineralization without a photocatalyst (Felix Gomez et al., 2018; Felix Gomez et al., 2019). Thus, light-only controls are essential when attributing antibacterial or anticaries effects to photocatalysis.

Visible-light sensitization through nitrogen doping can broaden activation conditions on exposed surfaces but cannot overcome absent illumination at inaccessible sites (Asahi et al., 2001).

Near-infrared upconversion offers a more experimental solution: NaYF_4_:Yb,Tm/TiO_2_ core-shell particles can convert 980-nm irradiation into shorter-wavelength emission that activates TiO_2_ (Mančić et al., 2023). This concept remains outside dentistry and has not established whether proposed selective effects persist under NIR excitation.

A pragmatic alternative is to restrict photocatalytic indications to readily illuminated sites, such as orthodontic appliances, sealants, removable prostheses, and anterior facial restorations. Patient-worn LED devices could extend exposure, but introduce compliance and have not been specifically evaluated for photocatalytic restorative materials.

Combining photocatalysis with constitutive activity may reduce dependence on illumination: silver- or copper-containing systems can add ion-mediated effects, and nitrogen-doped TiO_2_ has shown residual dark activity (Esteban Florez et al., 2018). Until irradiance is measured at clinically relevant sites, however, ‘light-activated’ should be regarded primarily as an experimental condition rather than an assured *in vivo* exposure state.

### Light activation, oral biofilm ecology, and selective inhibition

A 2022 AADOCR/CADR conference abstract proposed selective, light-induced inhibition of *S. mutans* by gold-titanate rather than nonspecific killing; because this evidence remains an abstract, it should be interpreted cautiously (Huang et al., 2022).

The reported system generated more hydrogen peroxide under dental LED activation and inhibited *S. mutans* while preserving tested commensal streptococci, conceptually mimicking peroxide-mediated ecological antagonism in oral biofilms (Huang et al., 2022).

This apparent selectivity remains preliminary because it was demonstrated in monoculture/co-culture experiments rather than intact multispecies biofilms and has not yet been independently replicated in a peer-reviewed full-length study.

### Evidence summary

**Table 4** groups the caries-relevant antibacterial evidence discussed above according to application—adhesive resins, orthodontic coatings, and titanate-based materials—and also summarizes the available antifungal evidence against *C. albicans*.

Photocatalytic titanium-based materials provide externally triggered ROS generation, but efficacy depends on light access and durable surface activity. Human evidence is limited to an orthodontic bacterial endpoint rather than caries prevention (Monica and Padmanabhan, 2022), and matched efficacy-toxicity data remain insufficient to define a robust therapeutic window (Chen et al., 2015; Zane et al., 2016; Esteban Florez et al., 2020a).

## Graphene-based materials

Graphene oxide (GO) and its derivatives, including formulations incorporating metal nanoparticles or nucleic acids, represent another class of antimicrobial materials with potential applications in caries prevention. Unlike the photocatalytic titanium-based materials discussed elsewhere in this review, which rely primarily on light-induced ROS, GO has an intrinsic physical antibacterial mechanism: its two-dimensional nanosheets have sharp edges that can disrupt bacterial membranes upon direct contact. The extent to which this activity has progressed toward clinical translation remains uncertain, as discussed in a recent review of graphene-based materials in caries and periodontal disease (Nizami et al., 2022).

### Mechanisms and caries-relevant antimicrobial evidence

GO’s antibacterial activity is generally attributed to two mechanisms that may operate concurrently: physical membrane disruption and oxidative stress. Sharp-edged GO nanosheets can damage the *S. mutans* envelope and cause cytoplasmic leakage (He et al., 2015), whereas ROS-mediated damage may affect bacterial DNA and proteins without requiring external light activation. Their relative contributions vary with synthesis and surface functionalization; for example, zinc oxide nanorods on graphene nanoplatelets acted predominantly through mechanical damage (Zanni et al., 2016), and oxidative activity has not consistently tracked with antibacterial potency in non-dental models (Liu et al., 2011). GO may also interfere with quorum sensing and adhesion and bind calcium at the enamel surface (Nizami et al., 2022). Quantitative antimicrobial concentrations and outcomes are summarized in **Table 5**.

**Table 5:** Antimicrobial evidence for graphene oxide and its derivatives in caries-relevant applications.

Material/system	Application	Target organism(s)	Effective conc.	Cytotoxic conc. tested	Key antimicrobial result	Ref.
Graphene oxide (GO) nanosheets	Antibacterial mechanism study	*S. mutans*	80 μg/mL	Not tested in this study; unmodified GO showed no toxicity <20 μg/mL but induced apoptosis >50 μg/mL in human fibroblasts (different, non-dental study) - 80 μg/mL antibacterial dose exceeds this cytotoxic threshold	83.7% viability reduction at 80 μg/mL; TEM shows membrane rupture and cytoplasmic leakage	(He et al., 2015)
ZnO nanorod-decorated graphene (ZNG)	Antibacterial nanomaterial	*S. mutans*	Not specified	N.A.	Antibacterial via mechanical membrane damage rather than ROS generation	(Zanni et al., 2016)
Graphene-ZnO nanocomposite (GZNC)	Coating on artificial acrylic tooth surfaces	*S. mutans*	Not specified	N.A.	LDH inhibition; lactic acid production −43%; biofilm thickness −62%; EPS −58%	(Kulshrestha et al., 2014)
GO-Ag nanocomposite (R-GNs/Ag)	Glass ionomer cement	*S. mutans*	1.00–2.00 wt%	No cytotoxicity data identified for this formulation (only mechanical properties were tested)	1.00–2.00 wt% significantly reduced viable *S. mutans*; flexural strength/microhardness not reduced up to 2.00 wt%	(Chen J. et al., 2020)
Reduced GO-Ag nanocomposite (rGO/Ag)	Artificial enamel caries model (*S. mutans* biofilm)	*S. mutans*	Not specified	N.A.	Shallower lesion depth, less mineral loss, smoother enamel surface vs. untreated control	(Wu et al., 2018)
GO-Cu nanocomposite	Biofilm-targeted antibacterial	*S. mutans*	10 μg/mL	Reported only as ‘low cytotoxicity’ at the 10 μg/mL antibacterial dose in the same study; no separate concentration-response cytotoxicity data given	10 μg/mL inhibits biofilm formation and EPS assembly; low cytotoxicity	(Mao et al., 2021)
GO-PEI-ASvicR (gene-silencing system)	Gene-silencing caries-management system	*S. mutans*	Not specified (only a cytotoxicity threshold is reported, ≤60 μg/mL)	≤60 μg/mL, low cytotoxicity reported in the same study (Wu et al., 2020)	Downregulates vicR-associated virulence genes; reduces EPS/biofilm; low cytotoxicity ≤60 μg/mL	(Wu et al., 2020)
Graphene nanocoating	Titanium surface coating	*C. albicans*	Not specified	N.A.	Persistent inhibition of biofilm formation and hyphae growth over extended culture	(Agarwalla et al., 2021)
GO-fluconazole compound	Antifungal combination therapy	*C. albicans* (fluconazole-resistant)	9–400 μg/mL (combination MIC; 800 μg/mL for GO alone)	N.A.	MIC reduced from 800 μg/mL (GO alone) to 9–400 μg/mL in combination	(Asadi Shahi et al., 2019)

MIC, minimum inhibitory concentration.

Much of the quantitative evidence against cariogenic organisms involves GO combined with a second antimicrobial component rather than unmodified GO. GO-ZnO, GO-Ag, and GO-Cu systems have reduced *S. mutans* metabolic activity, biofilm formation, EPS production, or viable counts, while some formulations have also shown preservation of material properties or reduced enamel mineral loss (Kulshrestha et al., 2014; Wu et al., 2018; Chen J. et al., 2020; Mao et al., 2021). Because these studies used different substrates and formulations, their concentrations and quantitative outcomes are summarized in **Table 5** rather than repeated in the text. Notably, the reduced-GO/Ag enamel model included a caries-relevant mineral endpoint, whereas several other studies relied primarily on microbiological surrogates (Kulshrestha et al., 2014; Wu et al., 2018; Chen J. et al., 2020; Mao et al., 2021).

A structurally distinct strategy uses GO as a delivery vehicle for genetic material rather than as the primary antimicrobial agent. GO complexed with polyethylenimine and an antisense vicR-expressing plasmid downregulated virulence-associated genes in *S. mutans*, reduced EPS synthesis, and impaired biofilm aggregation, with cytotoxicity evaluated in parallel (Wu et al., 2020). The tested concentrations are provided in **Table 5**.

GO studies remain dominated by *S. mutans* despite the polymicrobial nature of caries. Limited *C. albicans* evidence includes inhibition by graphene-coated titanium and additive activity of a GO-fluconazole formulation; a 2025 scoping review likewise noted broad antimicrobial signals but insufficient definitive toxicity and biocompatibility data (Asadi Shahi et al., 2019; Agarwalla et al., 2021; Martuci et al., 2025).

### Evidence summary

The caries-relevant antimicrobial evidence for GO and its derivatives discussed above is summarized in **Table 5**.

### Surface roughness and the antibacterial activity of GO

Because GO activity can depend on nanosheet edges, surface roughness is a relevant dental concern. Available studies do not show a simple trade-off: functionalized GO incorporated into denture acrylic up to tested levels did not significantly increase roughness, and other GO-containing dental materials retained acceptable surface characteristics while reducing microbial adhesion (Wang et al., 2011; Teimoorian et al., 2023; Moreira et al., 2024). Nevertheless, morphology, dispersion, and polishing can alter both antibacterial exposure and plaque-retentive surface properties, so these variables should be evaluated together.

Graphene-based materials remain primarily at the *in vitro* proof-of-concept stage. Their antimicrobial effects depend on surface contact and, in some formulations, oxidative mechanisms, while cytotoxicity varies with dose and surface functionalization (Wang et al., 2011; Gholami et al., 2020; Wu et al., 2020). Evidence for activity after salivary pellicle formation, selectivity among oral Gram-positive species, and direct anticaries efficacy remains limited; these gaps currently constrain translational interpretation.

## Zwitterionic polymers

Zwitterionic polymers contain no bactericidal payload. Although these hydrophilic materials carry both positive and negative charges within the same molecular structure, they are electrically neutral overall. Their potential for caries prevention therefore depends on preventing bacterial attachment rather than killing bacteria after attachment (Ramburrun et al., 2021). The two most widely studied zwitterionic polymers are 2-methacryloyloxyethyl phosphorylcholine (MPC) and sulfobetaine methacrylate (SBMA), with carboxybetaine-based polymers representing a third, less extensively studied family (Mangal et al., 2020). Because this is fundamentally an antifouling rather than bactericidal strategy, reductions in colony-forming units (CFUs) reported below primarily reflect reduced bacterial attachment, not increased bacterial killing.

### Antifouling mechanisms and caries-relevant antibiofilm evidence

The antifouling effect arises from a strongly hydrated layer at the material surface. The closely associated positive and negative charges organize water molecules and create an energetic barrier to protein adsorption, thereby limiting salivary pellicle formation before bacteria can attach (Mangal et al., 2020). An MPC-containing dental bonding agent reduced salivary protein adsorption by 95% and biofilm CFUs by four orders of magnitude compared with the control, while maintaining dentin bond strength (Zhang et al., 2014). Combining MPC with dimethylaminohexadecyl methacrylate (DMAHDM), a quaternary ammonium methacrylate, in a single resin composite couples protein repellency with contact-dependent killing. This combination is potentially advantageous because adsorbed proteins can shield bacteria from the surface and reduce the effectiveness of contact-active quaternary ammonium compounds. However, the proposed synergy has been demonstrated only *in vitro* and has not been evaluated against an aged, enzymatically remodeled salivary pellicle (Zhang et al., 2015; Torres and Bienek, 2020).

Carboxybetaine represents a third approach in which the antifouling chemistry is applied directly to tooth surfaces rather than incorporated into a polymerizable restorative matrix. A poly(carboxybetaine) polymer conjugated to catechol-based anchoring groups inhibited oral biofilm formation on both hydroxyapatite and human enamel without requiring polymerization (Sun et al., 2021).

Zwitterionic polymers have been incorporated into adhesives, composites, orthodontic materials, sealants, and denture resins, where they reduce protein adsorption and microbial attachment rather than directly kill bacteria (Zhang et al., 2015; Lee et al., 2020; Torres and Bienek, 2020; Kwon et al., 2021; Sun et al., 2021; Wu et al., 2023). Human studies reported reduced plaque accumulation or bacterial counts (Ikeya et al., 2018; Fujiwara et al., 2019), but these remain surrogate outcomes rather than evidence of reduced caries incidence. Formulation-specific results are summarized in **Table 6**.

**Table 6:** Antifouling and antibiofilm evidence for zwitterionic polymers in caries-relevant applications.

Material/system	Application	Study design/model	Target organism(s)	Effective conc.	Cytotoxic conc. tested	Key antifouling/antibacterial result	Ref.
MPC (2-methacryloyloxyethyl phosphorylcholine)	Dental bonding agent	*In vitro*	Salivary protein/general biofilm	Not specified	N.A.	95% reduction in protein adsorption; biofilm CFU 4-log lower than control; dentin bond strength preserved	(Zhang et al., 2014)
MPC + DMAHDM (quaternary ammonium methacrylate)	Dental resin composite	*In vitro*	General cariogenic biofilm	~3 wt% MPC (upper mechanically-compatible loading reported for this composite)	Not tested; cytotoxicity of the DMAHDM/QAC component is better characterized outside the zwitterionic literature, but no formulation-matched value was identified here.	Combined protein-repellent and antibacterial effect; mechanical properties comparable to BisGMA/TEGDMA up to ~3 wt% MPC	(Zhang et al., 2015)
Sulfobetaine methacrylate (SBMA)	Resin-based pit-and-fissure sealant	*In vitro*	General oral biofilm	Not specified	N.A.	Improved microbial resistance via reduced protein adsorption and bacterial adhesion	(Lee et al., 2020)
Zwitterionic derivative	Resin-modified glass ionomer luting cement	*In vitro*	General oral biofilm	Not specified (reported only as a concentration-dependent effect, no value given)	N.A.	Bacterial resistance conferred with concentration-dependent effect on physical properties	(Wu et al., 2023)
MPC or sulfobetaine derivatives	3D-printed PMMA denture base polymer	*In vitro*	Salivary biofilm	Not specified (described only as an “optimized concentration range”)	N.A.	Durable biofilm resistance maintained within optimized concentration ranges without mechanical compromise	(Kwon et al., 2021)
MPC-polymer coating	Complete denture surface (clinical)	Clinical (denture, parallel-group)	Denture plaque	Not specified	N.A.	Reduced plaque accumulation vs. uncoated dentures	(Ikeya et al., 2018)
MPC-polymer mouth rinse	Topical oral rinse (clinical, crossover)	Clinical (crossover)	General oral bacteria	Not specified	N.A.	Suppressed bacterial accumulation over several hours; caries-specific outcomes not assessed	(Fujiwara et al., 2019)
MPC, 1.5–4.5 wt%	High-impact denture base acrylic resin	*In vitro*	*C. albicans*	1.5–4.5 wt% MPC	N.A.	Reduced biofilm CFU relative to control, incorporated as a protein-repellent filler	(Bajunaid et al., 2021)
Photoreactive MPC surface coating	PMMA denture base coating	*In vitro*	*C. albicans*	Not specified	N.A.	Prevented biofilm formation; effect linked to salivary mucin interaction and retained after thermal cycling	(Putra Wigianto et al., 2023)
Zwitterionic-polymer grafted surface (vs. fluorinated surface)	Comparative antifouling coating	*In vitro* (comparative)	*C. albicans*	Not specified	N.A.	Reduced fungal adhesion; compared directly against a fluorinated non-zwitterionic alternative	(Masotti et al., 2020)

Clinical evidence for this class includes two independent human studies, which distinguishes zwitterionic polymers from most other material classes considered in this review. An MPC-polymer coating applied to complete dentures reduced plaque accumulation compared with uncoated controls, providing direct evidence that the antifouling effect can operate under intraoral conditions (Ikeya et al., 2018). In a separate study, an MPC-containing mouth rinse reduced bacterial accumulation over several hours compared with the control, although the outcome was bacterial burden rather than a caries-related clinical endpoint (Fujiwara et al., 2019). These findings therefore provide proof of concept for intraoral antifouling activity but do not establish that zwitterionic polymers reduce caries incidence.

Zwitterionic polymers also inhibit *C. albicans* through the same antifouling mechanism rather than through a distinct antifungal pathway. In several *in vitro* studies, MPC-containing denture-base resins and coatings reduced or prevented *C. albicans* biofilm formation compared with untreated and fluorinated controls (Masotti et al., 2020; Bajunaid et al., 2021; Putra Wigianto et al., 2023).

### Evidence summary

The caries-relevant antifouling and antibiofilm evidence for zwitterionic polymers discussed above is summarized in **Table 6**.

Zwitterionic polymers are mechanistically distinct because their principal function is antifouling rather than direct killing. Human studies have demonstrated reductions in plaque-related or bacterial outcomes, but not caries-specific endpoints (Ikeya et al., 2018; Fujiwara et al., 2019). Their favorable biocompatibility profile is encouraging, although oral dose-response data remain limited, and combination systems such as MPC-DMAHDM inherit safety considerations from the bactericidal component (Zhang et al., 2015; Mangal et al., 2020). The strong reduction in salivary protein adsorption reported for MPC-containing materials supports the rationale for this approach in the pellicle-rich oral environment (Zhang et al., 2014).

## Biomimetic nanozymes

Cariogenic biofilms are embedded within an extracellular polysaccharide (EPS) matrix that restricts molecular diffusion. This matrix can shield *S. mutans* from antimicrobial agents and confine acid production within localized microenvironments that are not readily accessible to mechanical plaque removal or fluoride-based therapies (Xu et al., 2025). Biomimetic nanozymes are engineered nanomaterials that mimic enzyme-like catalytic activity, including peroxidase-, oxidase-, catalase-, and superoxide dismutase-like functions (Liu et al., 2024). Their distinguishing feature is the ability of some formulations to become preferentially active within the biofilm microenvironment. For example, iron oxide catalytic nanoparticles (CAT-NP), in the presence of hydrogen peroxide, use peroxidase-like activity to degrade EPS and kill bacteria within acidic biofilm microenvironments (Gao et al., 2016).

### Mechanisms and caries-relevant antimicrobial evidence

Peroxidase-like catalysis is the principal mechanism underlying the iron oxide platform. Under the acidic conditions of cariogenic biofilms, iron oxide nanoparticles catalyze the conversion of hydrogen peroxide into ROS, thereby promoting bacterial killing while simultaneously degrading the surrounding EPS matrix (Gao et al., 2016). A dextran-coated iron-oxide (Fe_3_O_4_) nanozyme (Dex-NZM) further enhances biofilm localization (Naha et al., 2019). Selectivity can be increased by combining glucose oxidase with dextran-coated iron oxide nanoparticles (Dex-IONP-GOx). This system generates hydrogen peroxide from glucose within the biofilm and subsequently converts it to ROS, suppressing *S. mutans* while sparing the commensal species *S. oralis*. The reported selectivity reflects both dextran-mediated binding to *S. mutans*-associated glucan-binding proteins and the lower ROS tolerance of the pathogen relative to the commensal species (Huang et al., 2021).

The same general principle has been explored in several other systems. These include a magnetic cobalt-platinum-graphene nanohybrid (Dong et al., 2022), a system that uses hydrogen peroxide generated by the commensal species *S. gordonii* (Wang et al., 2020), and an ultrasound-responsive platform in which ultrasound promotes peroxide release, followed by Fenton chemistry that generates hydroxyl radicals (Guo et al., 2023). Cerium oxide nanozymes operate through a different mechanism, using Ce^3+^/Ce^4+^ redox cycling to scavenge ROS rather than generate them, particularly under physiological pH conditions (Dhall and Self, 2018).

The original CAT-NP study demonstrated both antibacterial activity and EPS degradation, with bacterial killing and matrix disruption occurring at the same biofilm sites (Gao et al., 2016). A major translational step was the use of ferumoxytol, an iron oxide nanoparticle already approved by the U.S. Food and Drug Administration (FDA) for intravenous treatment of iron deficiency. In combination with low-concentration hydrogen peroxide, ferumoxytol disrupted biofilm architecture and killed embedded bacteria. Subsequent topical administration reduced severe cavitated lesions *in vivo* without detectable changes in microbiota diversity or injury to gingival or mucosal tissues (Liu et al., 2018).

A subsequent randomized crossover human *in situ* study provided evidence that the platform can suppress *S. mutans*-rich biofilms under intraoral conditions. Ferumoxytol combined with hydrogen peroxide preferentially bound to and suppressed *S. mutans*-rich biofilms relative to commensal streptococci. Its peroxidase-like catalytic activity also provided a visual readout of pathogenic biofilm sites. The reported selectivity was attributed not only to the acidic, peroxide-generating biofilm microenvironment but also to direct recognition of the dextran coating by glucan-binding proteins associated with *S. mutans* (Liu et al., 2021). The hydrogen peroxide concentrations evaluated in these studies overlap with those used in some commercial oral products (Liu et al., 2018). However, this concentration overlap alone does not establish the safety of repeated topical use in combination with a catalytic nanozyme, which may alter the magnitude and localization of ROS generation.

A more recent development combines Fe_3_O_4_ nanozymes with stannous fluoride (SnF_2_), with the two components providing complementary effects. The combination produced more than a 5-log reduction in viable *S. mutans*, promoted deposition of a protective iron-tin-fluoride layer on enamel, and prevented cavitation *in vivo* at an SnF_2_ dose fourfold lower than that required for SnF_2_ alone (Huang et al., 2023). A structurally distinct silver-graphdiyne ointment uses another approach: under acidic plaque conditions, it converts low concentrations of hydrogen peroxide into hydroxyl radicals while simultaneously releasing Ag^+^ ions. The formulation also promotes salivary calcium and phosphate deposition, thereby supporting hydroxyapatite formation (Liao et al., 2024).

The selectivity observed with Dex-IONP-GOx also translated into reduced disease severity *in vivo*. Topical treatment significantly reduced caries severity across all lesion categories compared with control treatment or either component alone and was more effective than chlorhexidine for the most severe, fully cavitated lesions (Huang et al., 2021).

Evidence that iron oxide nanozymes directly inhibit *C. albicans* remains limited. To date, it consists primarily of a supplementary finding from the ferumoxytol rodent study (Liu et al., 2018) and a significant reduction in *C. albicans* CFU reported for a separate peroxidase-mimicking cobalt ferrite–graphene oxide nanozyme (Ghaffari et al., 2026). The most comprehensive antifungal evidence comes from a different approach in which chitosan-coated iron oxide nanoparticles served solely as drug-delivery carriers for conventional antifungal agents rather than relying on intrinsic nanozyme activity (Caldeirão et al., 2021).

### Evidence summary

**Table 7** summarizes the antibacterial and antifungal evidence discussed above and indicates whether the available *C. albicans* evidence is direct, preliminary, or derived from a mechanistically distinct, delivery-based system.

**Table 7:** Antibacterial and antifungal evidence for biomimetic nanozymes in caries-relevant applications.

Material/system	Application	Target organism(s)	Effective conc.	Cytotoxic conc. tested	Key result	Pathogen vs. commensal compared?	Ref.
CAT-NP (Fe_3_O_4_) + H_2_O_2_	*In vitro* biofilm model (saliva-coated hydroxyapatite discs); *in vivo* rodent caries model	*S. mutans*	Not specified	Tested in BJ5ta fibroblasts - no overt cytotoxicity reported, but exact concentration not stated	EPS-matrix degradation and bacterial killing concentrated at acidic pH; biocompatible with BJ5ta fibroblasts *in vitro*	No	(Gao et al., 2016)
Dex-NZM (dextran-Fe_3_O_4_) + H_2_O_2_, pH ≈ 4.5	*In vitro* mixed biofilm; *in vivo* rodent caries model	*S. mutans* (mixed biofilm)	Not specified (pH ~4.5 activation condition only, no dose given)	N.A.	Up to 6-log reduction in viable bacterial counts; reduced biofilm biomass; lower caries severity *in vivo* without gingival or microbiota harm	No	(Naha et al., 2019)
Ferumoxytol (FDA-approved iron oxide nanoparticle) + H_2_O_2_	*Ex vivo* human biofilm on natural teeth; *in vivo* rodent model	*S. mutans*-dominant biofilm	Not specified in this study; related ferumoxytol work reported activity with H2O2 within the clinically used 1.5-10% range.	No *in vitro* cytotoxicity concentration reported; *in vivo*, no adverse effect on gingival tissue or microbiota diversity observed	Biofilm disruption via membrane damage and EPS degradation; prevented severe cavitation *in vivo*; no adverse effect on gingival tissue or microbiota diversity	No	(Liu et al., 2018)
Dex-IONP-GOx (dual-catalytic nanohybrid)	*In vitro* mixed-species biofilm (*S. mutans* + *S. oralis*); *in vivo* rodent caries model	*S. mutans* (selective)	Not specified (efficacy reported as log-reduction, not dose)	Clinically relevant concentration range (exact value not stated); no adverse effect on human gingival epithelial cell/fibroblast viability - same study/material	Selectively killed *S. mutans* (>7-log reduction in 3 h) while sparing commensal *S. oralis* (<1-log change); FISH-supported specificity; selectivity driven by preferential binding plus differential ROS tolerance; disrupted EPS 3-D architecture; *in vivo*, reduced caries severity at all lesion stages and outperformed chlorhexidine on cavitation severity and microbiota preservation	Yes (vs. *S. oralis*)	(Huang et al., 2021)
Ferumoxytol + H_2_O_2_	Randomized crossover human intraoral enamel-specimen study	*S. mutans*-rich biofilm in situ	Not specified in this study; related ferumoxytol work reported activity with H2O2 within the clinically used 1.5-10% range.	No cytotoxicity concentration reported for this study; no gingival/mucosal damage or microbiota disruption observed in related ferumoxytol work	Preferential binding to and suppression of *S. mutans* over commensal streptococci in the human mouth; TMB colorimetric reaction enabled visual biofilm detection	Yes (vs. commensal strep.)	(Liu et al., 2021)
Fe_3_O_4_ nanozyme + SnF_2_	*In vivo* rodent model, topical application	*S. mutans*	Not specified (SnF_2_ dose reported only as four-fold lower than SnF_2_ alone, no absolute value given)	N.A.	Greater than 5-log reduction in viable *S. mutans*; co-deposited Fe–Sn–F enamel layer; prevented cavitation at four-fold lower SnF_2_ dose than SnF_2_ alone	No	(Huang et al., 2023)
Ferumoxytol + H_2_O_2_ (preliminary, supplementary data)	Exploratory *ex vivo/in vitro*/rodent assay, not a powered endpoint	*C. albicans*	Not specified; related ferumoxytol work reported activity with H2O2 within the clinically used 1.5-10% range.	No cytotoxicity concentration reported; no gingival/mucosal damage or microbiota disruption observed in related ferumoxytol work	Preliminary antifungal signal (P < 0.05–0.001) alongside *S. gordonii*-mediated ecological suppression of *S. mutans*	No	(Liu et al., 2018)
Chitosan-coated iron oxide nanoparticles carrying miconazole or fluconazole	Saliva-derived oral microcosm biofilm	*C. albicans*, *C. glabrata*	Not specified	N.A.	Both nanocarriers improved *C. albicans* CFU reduction vs. drug alone; clearer *C. glabrata* advantage seen mainly with the miconazole carrier; mechanism is drug delivery, not intrinsic nanozyme catalysis	No	(Caldeirão et al., 2021)
CoFe_2_O_4_/GO@Dex-CS nanozyme + H_2_O_2_	*In vitro* six-species oral biofilm model	*C. albicans*	Not specified	N.A.	Peroxidase-mimicking dextran/chitosan-coated cobalt ferrite-graphene oxide nanozyme produced significant *C. albicans* CFU reduction; fungal-specific mechanisms, host selectivity, and *in vivo* efficacy not evaluated	No	(Ghaffari et al., 2026)
CoPt@graphene@GOx nanohybrid	*In vitro* planktonic and biofilm assay	Mixed oral bacteria	Not specified	N.A.	GOx-generated H_2_O_2_ converted to hydroxyl radicals by CoPt@graphene peroxidase mimic; magnetically actuable; effective bactericidal activity in both planktonic and biofilm states	No	(Dong et al., 2022)
Iron oxide + iron sulfide nanozymes with *S. gordonii* (commensal H_2_O_2_ source)	*In vitro* biofilm on human dentin surfaces	*S. mutans*	Not specified	N.A.	Commensal-generated H_2_O_2_ sustained peroxidase-like catalysis without exogenous H_2_O_2_ addition; reduced *S. mutans* biofilm formation on dentin	No	(Wang et al., 2020)
Ultrasonically activated microbomb (CaO_2_-tannic acid-iron/perfluorohexane/PLGA)	*In vitro* dental biofilm; *in vivo* rat caries model, ultrasonic-toothbrush-triggered	Dental biofilm (mixed flora)	Not specified	N.A.	Ultrasound-triggered phase shift releases CaO_2_-derived H_2_O_2_; tannic acid-iron network converts it to hydroxyl radicals via Fenton chemistry; physical rather than chemical/biological trigger	No	(Guo et al., 2023)
Graphdiyne/L-cysteine/Ag (GLA) nanozyme ointment	Topical ointment, acid-activated; *in vivo* rodent caries model	Cariogenic biofilm; enamel remineralization	Not specified (described only as “low-dose” H_2_O_2_ and Ag^+^ release)	N.A.	Converts low-dose H_2_O_2_ into hydroxyl radicals under acidic plaque conditions (synergistic with Ag^+^ release); separately nucleates calcium and phosphate ions from saliva to promote hydroxyapatite growth	No	(Liao et al., 2024)

Several questions remain unresolved. It is not yet clear whether the pathogen-selective activity demonstrated against bacteria extends to *C. albicans* with comparable evidence. Independent replication is also needed, particularly because much of the available evidence comes from a limited number of research groups. Finally, whether this approach can be incorporated into a durable delivery format suitable for longer intraoral exposure, rather than short-contact topical application, remains to be determined.

Nanozymes provide microenvironment-responsive catalytic activity and therefore represent one of the more mechanistically distinctive classes reviewed. Ferumoxytol-based systems have reached randomized *in situ* evaluation of biofilm suppression, but not a direct clinical caries endpoint (Liu et al., 2021). Reported host tolerance is encouraging, yet intravenous approval of ferumoxytol does not establish topical oral safety, and broader independent validation of oral formulations remains necessary (Gao et al., 2016; Liu et al., 2018; Liu et al., 2021).

## Antimicrobial peptides: C16G2 and other caries-relevant peptides

Antimicrobial peptides typically act through membrane disruption, although caries-directed designs increasingly add pathogen-targeting or virulence-modulating functions (Ying et al., 2023). C16G2 is antimicrobial peptide that couples an *S. mutans*-targeting domain to a broad-spectrum killing domain, thereby concentrating membrane-disruptive activity on the target organism (Eckert et al., 2006b; Eckert et al., 2006a; He et al., 2010; Li et al., 2010; Kaplan et al., 2011; Eckert et al., 2012).

Beyond C16G2, representative peptides illustrate broader design strategies. GH12 suppresses *S. mutans* growth, biofilm formation, acidogenicity, aciduricity, EPS-associated virulence, multispecies biofilms, and caries in an animal model (Wang et al., 2017; Jiang et al., 2018; Wang Y. et al., 2018; Jiang et al., 2020). GA-C16G2 extends the C16G2 scaffold through gallic-acid conjugation (Fu et al., 2025); P-113-DPS suppresses *S. mutans* cariogenic virulence (Liu et al., 2025); PMF was designed for selective antibacterial and antibiofilm activity against *S. mutans* (Wei et al., 2026); and ZXR-2 provides additional *in vitro* activity against caries-associated bacteria (Chen L. et al., 2017).

Together, these studies show a shift toward pathogen-targeted, virulence-modulating, and ecologically informed peptide designs (Jiang et al., 2026). Most non-C16G2 candidates remain preclinical, with proteolytic stability, microbial selectivity, host-cell safety, manufacturing cost, and standardized clinical evaluation remaining key barriers (Jiang et al., 2026).

### Mechanism and caries-relevant preclinical and clinical evidence

C16G2 is more potent against *S. mutans* than its isolated killing domain and shows marked selectivity over *S. gordonii* and *S. sanguinis*, indicating that specificity is conferred by the targeting domain (Eckert et al., 2006a). In saliva-derived mixed-species biofilms, it rapidly eliminated *S. mutans* with little measurable effect on these commensals (Eckert et al., 2006a).

C16G2 kills through selective membrane depolarization and disruption, producing rapid reductions in *S. mutans* while largely sparing non-target streptococci (Kaplan et al., 2011). Repeated sub-inhibitory passage did not increase its minimum inhibitory concentration (MIC), and pretreatment reduced subsequent *S. mutans* recolonization (He et al., 2010; Li et al., 2010), although these findings require independent replication.

The only peer-reviewed clinical publication is a pilot study in which a 0.04% mouth rinse reduced plaque and salivary *S. mutans*, lactic acid production, and enamel demineralization with little change in total plaque bacteria (Sullivan et al., 2011; ClinicalTrials.gov, 2022a). The abstract does not report sample size, effect sizes, or p-values, limiting quantitative interpretation.

C16G2 subsequently entered four registered Phase 2 trials evaluating varnish, strip, combination regimens, or gel; three posted results in 2022 and one has not reported results (ClinicalTrials.gov, [[NoYear]]; ClinicalTrials.gov, 2022a; ClinicalTrials.gov, 2022b; ClinicalTrials.gov, 2022c). In the completed trials, changes in salivary or plaque *S. mutans* were comparable to or smaller than placebo, without formal statistical comparisons for the primary microbiological endpoint (ClinicalTrials.gov, 2022a; ClinicalTrials.gov, 2022b; ClinicalTrials.gov, 2022c). Details are summarized in **Table 8**.

**Table 8:** Representative antibacterial, antibiofilm, and clinical evidence for C16G2 and other caries-relevant antimicrobial peptides.

Material/system	Application	Target organism(s)	Effective conc.	Cytotoxic conc. tested	Key result	Ref.
C16G2 (parent STAMP peptide)	Mechanism/MIC-MBC characterization	*S. mutans* vs. *S. gordonii*, *S. sanguinis*	MIC 3–5 µM	Not independently tested for the parent molecule (see GA-C16G2* below)	MIC 3–5 µM vs. 12–20 µM for the killing domain alone; >20-fold greater 1-min activity against *S. mutans* at equal potency against commensals	(Eckert et al., 2006a)
C16G2, 3.125–6.25 µM	Membrane-disruption mechanism study	*S. mutans* vs. *S. gordonii*, *S. sanguinis*, *S. salivarius*	3.125–6.25 µM	N.A.	>2 log10 CFU/mL reduction in *S. mutans* at 10 min (P = 0.01) vs. 47% reduction in non-*S. mutans* streptococci; SYTOX/DiSC3 (Kopperud et al., 2012) confirm selective membrane depolarization	(Kaplan et al., 2011)
C16G2, sub-MIC serial passage	Resistance-development study	*S. mutans*	Sub-MIC (exact concentration not specified)	Not applicable (resistance assay, not a cytotoxicity study)	No MIC increase reported over repeated sub-inhibitory passage, in contrast to a streptomycin-resistant control strain	(He et al., 2010)
STAMP-pretreated saliva-derived biofilm	*Ex vivo* colonization-resistance model	*S. mutans* in pooled human saliva biofilm	Not specified in *ex vivo* protocol	N.A.	STAMP pretreatment reduced subsequent *S. mutans* recolonization of an established non-cariogenic biofilm	(Li et al., 2010)
C16G2, 0.04% (w/v) mouth rinse	Clinical pilot, intra-oral remineralization model	Human subjects (*S. mutans*, plaque, enamel)	0.04% w/v	N.A.	Rinse use associated with reduced plaque/salivary *S. mutans*, lactic acid production, and demineralization; minimal effect on total plaque bacteria (exact n/p not available from abstract)	(Sullivan et al., 2011)
C16G2 varnish, 13.6/27.2/54.4 mg	Phase 2 RCT, dose-ranging (NCT03004365, n=33)	Human subjects, salivary/plaque *S. mutans* CFU	13.6–54.4 mg/dose	None reported (zero drug-related adverse events across n=33)	Mean log CFU/mL change from baseline comparable to or smaller than pooled placebo at every dose tested; no p-values posted	(ClinicalTrials.gov, 2022a)
C16G2 strip, 9.2/18.4/36.8 mg	Phase 2 RCT, dose-escalation (NCT03052842, n=30)	Human subjects, salivary *S. mutans* CFU	9.2–36.8 mg/dose	None reported (zero drug-related adverse events across n=30)	Near-zero to modest CFU change across all three doses, smaller in magnitude than the placebo arm’s reduction; no p-values posted	(ClinicalTrials.gov, 2022b)
C16G2 varnish + strip	Phase 2 RCT, combination regimen (NCT03196219, n=20)	Human subjects, salivary *S. mutans* CFU	Varnish + strip, doses per component trials	None reported (zero drug-related adverse events across n=20)	Varnish-alone arm showed a CFU increase from baseline; placebo arm showed a larger reduction than active varnish; no p-values posted	(ClinicalTrials.gov, 2022c)
C16G2, all Phase 2 arms combined	Safety/tolerability pooled analysis (n=83 active+placebo)	Human subjects, adverse events	Not applicable (safety-focused analysis)	Zero deaths, zero serious adverse events, zero drug-related adverse events, 100% completion	Zero deaths, zero serious adverse events, zero drug-related adverse events, 100% completion across all reporting arms	(ClinicalTrials.gov, 2022a; ClinicalTrials.gov, 2022b; ClinicalTrials.gov, 2022c)
GA-C16G2 (gallic acid conjugate)	Antibacterial/cytotoxicity characterization	*S. mutans*; HGF-1 human gingival fibroblasts	MIC 3.1 µM, MBC 20.8 µM	Minimal cytotoxicity across 3.125–400 µM (~100× the MIC)	MIC 3.1 µM, MBC 20.8 µM; minimal HGF-1 cytotoxicity across 3.125–400 µM (~100× the MIC)	(Fu et al., 2025)
GH12	Preclinical antibacterial/anticaries studies	*S. mutans*; multispecies cariogenic biofilms	Study-dependent	Preclinical	Suppressed *S. mutans* virulence and cariogenic multispecies biofilm properties; reduced caries in a rat model	(Wang et al., 2017; Jiang et al., 2018; Wang Y. et al., 2018; Jiang et al., 2020)
P-113-DPS	Virulence-modulating AMP study	*S. mutans*	Study-dependent	*In vitro*	Reduced growth, acidogenicity, aciduricity, EPS synthesis, biofilm formation, and virulence-associated gene expression	(Liu et al., 2025)
PMF dual-targeting peptide	Species-selective antibacterial/antibiofilm study	*S. mutans* vs*. S. sanguinis* and *S. gordonii*	MIC/MBC 1.56/3.13 µM	*In vitro* safety testing included	Potent *S. mutans* killing and biofilm disruption with markedly lower activity against tested commensal streptococci	(Wei et al., 2026)
ZXR-2	Antibacterial/antibiofilm characterization	Caries-associated oral bacteria; *S. mutans*	Study-dependent	Preclinical	Rapid antibacterial activity and inhibition of *S. mutans* biofilm formation	(Chen L. et al., 2017)

* GA-C16G2, Gallic acid-C16G2; STAMP, specifically targeted antimicrobial peptide; MIC, minimum inhibitory concentration; MBC, minimum bactericidal concentration.

These small exploratory dose-finding studies did not establish a clear, statistically supported dose-response advantage over placebo, but they were not definitive efficacy trials. The appropriate interpretation is therefore failure to establish efficacy, rather than evidence that C16G2 is ineffective.

The corresponding sample-size and formulation details are summarized in **Table 8**.

### Evidence summary

**Table 8** summarizes representative mechanistic, preclinical, and clinical evidence for C16G2 and other caries-relevant antimicrobial peptides.

Among the peptides reviewed, C16G2 has the most advanced human evidence, although completed Phase 2 studies did not establish a clear advantage over placebo (ClinicalTrials.gov, [[NoYear]]; Sullivan et al., 2011; ClinicalTrials.gov, 2022a; ClinicalTrials.gov, 2022b; ClinicalTrials.gov, 2022c; Namburu et al., 2022). GH12 has a broader preclinical chain spanning virulence, multispecies biofilms, plaque ecology, and animal caries outcomes (Wang et al., 2017; Jiang et al., 2018; Wang Y. et al., 2018; Jiang et al., 2020), whereas GA-C16G2, P-113-DPS, PMF, and ZXR-2 remain primarily laboratory-supported (Chen L. et al., 2017; Fu et al., 2025; Liu et al., 2025; Wei et al., 2026). Clinical anticaries efficacy beyond C16G2 remains unestablished (Jiang et al., 2026), and no published study has assessed C16G2 compatibility with restorative materials.

Despite being the most clinically advanced peptide reviewed, C16G2 has not demonstrated clinical anticaries efficacy, and the remaining peptide candidates are predominantly preclinical. Future development should prioritize ecological selectivity, oral stability, host safety, durable delivery, and clinically meaningful caries endpoints (ClinicalTrials.gov, [[NoYear]]; Eckert et al., 2006a; Sullivan et al., 2011; ClinicalTrials.gov, 2022a; ClinicalTrials.gov, 2022b; ClinicalTrials.gov, 2022c; Fu et al., 2025; Jiang et al., 2026).

## Additional strategies outside the seven-class framework

Four additional strategies merit brief mention outside the seven-class framework. Bioactive glass releases mineral ions, raises local pH, and promotes remineralization; its antibacterial activity against aciduric organisms such as *S. mutans* appears secondary to alkalinization, and the clinical contribution of antimicrobial versus mineral effects remains difficult to separate (Waltimo et al., 2007; Dai et al., 2019; Skallevold et al., 2019). pH-responsive polymeric carriers can preferentially release agents such as farnesol under cariogenic acidic conditions and have reduced biofilm virulence in a rodent model, but human and restorative-material evidence is lacking (Horev et al., 2015; He et al., 2023). Covalently bound organoselenium can generate superoxide through thiol oxidation and has inhibited bacterial or *C. albicans* biofilms in dental materials, including clinical evaluations around sealants and orthodontic brackets; long-term caries-outcome evidence remains limited (Kramer and Ames, 1988; Chaudiere et al., 1992; Seko and Imura, 1997; Tran et al., 2013; Tran et al., 2014; Amaechi et al., 2018; Amaechi et al., 2019b; Amaechi et al., 2021; AlMojel et al., 2023; Alawadi et al., 2024). CRISPR-based antimicrobials add sequence-level precision: *S. mutans* glucosyltransferase editing reduced EPS and biofilm formation, Cas13a established sequence-specific bacterial killing, and nanoparticles have been proposed as delivery carriers (Gong et al., 2018; Kiga et al., 2020; Saffari Natanzi et al., 2025). Most recently, a probiotic-vesicle CRISPR platform targeting *gtfB* combined *S. mutans* suppression, reduced biofilm/caries severity, and microbiome modulation in a preclinical model (Zhou et al., 2026). Delivery, off-target effects, carrier reproducibility, host responses, and regulation remain major translational barriers.

## Where the evidence base runs thin

Across the seven classes, recurring limitations include heterogeneous outcome measures, limited clinical translation, concentration of evidence within a small number of research groups, and formulation-dependent trade-offs between antimicrobial activity and material performance.

Claims of selectivity are often supported by narrow organism panels. Graphene studies focus predominantly on *S. mutans*, with little commensal comparison and no basis for inferring selectivity among clinically relevant Gram-positive streptococci from peptidoglycan thickness alone (Pulingam et al., 2019). Gold-titanate selectivity has not been tested in intact multispecies biofilms (Huang et al., 2022). Nanozyme antifungal evidence is preliminary or formulation-specific, and cerium-oxide relevance to caries remains indirect (Dhall and Self, 2018; Liu et al., 2018; Caldeirão et al., 2021; Zhang et al., 2024; Ghaffari et al., 2026). For zwitterionic polymers, reduced CFU may reflect impaired attachment rather than killing; intraoral testing of proposed zwitterionic-QAC combinations and durability evidence also remain limited (Torres and Bienek, 2020; Putra Wigianto et al., 2023).

Clinical endpoints are another major gap. Human zwitterionic studies measured plaque or bacterial outcomes rather than caries (Ikeya et al., 2018; Fujiwara et al., 2019), whereas randomized nano silver fluoride trials directly evaluated caries arrest (Nagireddy et al., 2019; Quritum et al., 2024). A broad systematic review found no established clinical anticaries efficacy for restorative-incorporated antimicrobial materials (do Amaral et al., 2016). C16G2 illustrates the converse problem: despite extensive clinical development, completed Phase 2 microbiological outcomes were modest and lacked formal statistical comparisons, with no conventional animal caries model or clinical caries-incidence trial reported (ClinicalTrials.gov, [[NoYear]]; Armata Pharmaceuticals I, 2020; ClinicalTrials.gov, 2022a; ClinicalTrials.gov, 2022b; ClinicalTrials.gov, 2022c; Fu et al., 2025). Thus, human testing based only on plaque or bacterial endpoints should not be equated with demonstrated anticaries efficacy.

Overall, antimicrobial findings remain much more common than direct anticaries outcomes (do Amaral et al., 2016). Methodological heterogeneity further limits comparison; ISO 3990:2023 standardizes antibacterial testing for several dental material categories but does not establish clinical caries prevention (International Organization for Standardization, 2023). Multifunctional combinations should therefore be tested with standardized methods and clinically relevant endpoints (Melo et al., 2023).

**Table 9** provides a concise cross-class comparison of predominant mechanism, trigger, furthest translational stage, compatibility cost, and esthetic risk. Translational stage is classified by the endpoint actually measured, distinguishing microbiological human studies from caries-specific trials.

**Table 9:** Cross-class comparison of the seven antimicrobial material classes surveyed in this review.

Material class	Antimicrobial mechanism	Trigger	Furthest translational stage reached	Main translational/compatibility limitation	Esthetic risk
QAC/QPEI	Cationic contact-mediated membrane disruption	Constitutive	*In situ* biofilm exposure (QPEI); predominantly *in vitro* evidence for polymerizable QAC monomers; no caries-outcome RCT for either subclass	QPEI: dose-dependent flexural strength/water sorption cost; monomeric family: minimal within tested ranges	Not addressed as a distinct question in the literature reviewed
Metallic Nanoparticles (Ag, ZnO)	Ion release + contact-mediated membrane disruption/ROS	Constitutive	RCT, caries arrest (nano silver fluoride, topical only); restorative-incorporated applications remain *in vitro*	Interfacial shielding and pellicle passivation limit incorporated (vs. topical) restorative use	Lower for topical nano silver fluoride than for silver diamine fluoride (reduced tooth discoloration in head-to-head RCT); not addressed for restorative-incorporated Ag/ZnO formulations
Photocatalytic TiO_2_	Light-driven reactive-oxygen generation	External light	RCT, bacterial/plaque endpoint (orthodontic brackets only); caries-incidence endpoint not tested	Light-delivery geometry restricts viable clinical sites more than material compatibility does	Dopant-dependent rather than uniform: undoped TiO_2_ is white/opaque, nitrogen doping imparts a yellow tint, and metal dopants (Ag, Cu, Au) give grey-brown to plasmonic coloration
Graphene-Based Materials (GO/rGO/GNP composites)	Physical nanosheet puncture + oxidative stress	Constitutive	*In vitro* only; no rodent or human model identified	Mixed: improves some resin/GIC formulations, reduces flexural modulus in one CAD/CAM PMMA system	High: intrinsic dark pigmentation limits use to thin, low-concentration, non-visible layers
Zwitterionic Polymers	Antifouling hydration barrier (non-bactericidal)	Constitutive/passive	Clinical trial, plaque/bacterial endpoint (denture coating, mouth-rinse); caries-incidence endpoint not tested	Concentration-dependent mechanical cost above roughly 3 wt% MPC	Not addressed as a distinct question in the literature reviewed
Biomimetic Nanozymes	Enzyme-mimetic catalysis (peroxidase-like ROS generation)	Biofilm microenvironment (acidity, H_2_O_2_, glucose)	*In situ* biofilm, randomized crossover design (ferumoxytol); caries-incidence endpoint not tested	Not yet tested for retained catalytic activity after incorporation into a restorative matrix	Fe_3_O_4_-SnF_2_ coating’s enamel discoloration risk not yet evaluated
Antimicrobial Peptides (C16G2 as the clinically most advanced example)	Peptide-mediated membrane disruption, pathogen targeting, and/or virulence modulation, depending on peptide design	Peptide-dependent; constitutive or bioresponsive	C16G2: pilot clinical study with a caries-relevant endpoint; 4 registered Phase 2 trials, 3 with posted results (bacterial-CFU endpoint); other peptides remain preclinical	Most candidates remain preclinical; oral stability, selectivity, host safety, durable delivery, and restorative-material compatibility require further study	Not identified as a major class-wide limitation in the literature reviewed

## Conclusion

No single class reviewed here resolves the competing demands of antimicrobial activity, material compatibility, safety, ecological selectivity, and clinical caries prevention. QACs and metallic nanoparticles have comparatively mature restorative-incorporation evidence, whereas photocatalytic TiO_2_ and nanozymes offer triggered catalytic mechanisms but remain constrained by activation conditions and limited independent validation. Zwitterionic polymers emphasize antifouling rather than killing, graphene remains largely preclinical, and antimicrobial peptides illustrate the potential and difficulty of species-targeted approaches. C16G2 progressed furthest clinically, yet its completed Phase 2 program did not establish a clear advantage over placebo, and no subsequent late-stage clinical development was identified in the literature reviewed (ClinicalTrials.gov, [[NoYear]]; Sullivan et al., 2011; Armata Pharmaceuticals I, 2020; ClinicalTrials.gov, 2022a; ClinicalTrials.gov, 2022b; ClinicalTrials.gov, 2022c; Fu et al., 2025). Across classes, microbiological suppression remains far better documented than reduced demineralization, lesion progression, or caries incidence. Future translation therefore requires clinically relevant anticaries endpoints and formulation-specific therapeutic windows that demonstrate antimicrobial benefit at exposures below unacceptable host toxicity; for several classes, especially QACs and metallic nanoparticles, current efficacy and cytotoxicity datasets are too heterogeneous to define that margin reliably (Tabari et al., 2017; Szymczak-Pajor et al., 2025).

## References

[B66] Abdulrazzaq Naji S. Jafarzadeh Kashi T. S. Pourhajibagher M. Behroozibakhsh M. Masaeli R. Bahador A. (2018). Evaluation of antimicrobial properties of conventional poly(methyl methacrylate) denture base resin materials containing hydrothermally synthesised anatase TiO(2) nanotubes against cariogenic bacteria and Candida albicans. Iran. J. Pharm. Res. 17, 161–172. doi: 10.22037/ijpr.2018.2362 PMC6447881 31011350

[B32] Abramovitz I. Beyth N. Paz Y. Weiss E. I. Matalon S. (2013). Antibacterial temporary restorative materials incorporating polyethyleneimine nanoparticles. Quintessence Int. 44, 209–216. doi: 10.3290/j.qi.a29056 23444202

[B100] Agarwalla S. V. Ellepola K. Silikas N. Castro Neto A. H. Seneviratne C. J. Rosa V. (2021). Persistent inhibition of Candida albicans biofilm and hyphae growth on titanium by graphene nanocoating. Dent. Mater. 37, 370–377. doi: 10.1016/j.dental.2020.11.028 33358443

[B68] Ahmed A. Q. Al-Hmedat S. J. A. Hanweet D. M. Haider J. (2023). Assessing the antifungal activity of a soft denture liner loaded with titanium oxide nanoparticles (TiO2 NPs). Dent. J. (Basel) 11, 90. doi: 10.3390/dj11040090 37185468 PMC10137426

[B168] Alawadi A. AbdulAzees P. A. Lin C. Y. Haney S. J. Hanlon J. P. Angelara K. . (2024). Application of organoselenium in inhibiting Candida albicans biofilm adhesion on 3D printed denture base material. J. Prosthodont. 33, 460–466. doi: 10.1111/jopr.13733 37422719

[B40] Aldulaijan S. Alruwili R. Almulaify R. Alhassan F. A. Al-Dulaijan Y. A. Alshahrani F. A. . (2024). Benzyldimethyldodecyl ammonium chloride-doped denture-based resin: Impact on strength, surface properties, antifungal activities, and in silico molecular docking analysis. J. Funct. Biomater. 15, 310. doi: 10.3390/jfb15100310 39452608 PMC11508443

[B167] AlMojel N. AbdulAzees P. A. Lamb E. M. Amaechi B. T. (2023). Determining growth inhibition of Candida albicans biofilm on denture materials after application of an organoselenium-containing dental sealant. J. Prosthet. Dent. 129, 205–212. doi: 10.1016/j.prosdent.2021.04.015 34078547

[B69] Altarazi A. Jadaan L. McBain A. J. Haider J. Kushnerev E. Yates J. M. . (2024). 3D-printed nanocomposite denture base resin: the effect of incorporating TiO(2) nanoparticles on the growth of Candida albicans. J. Prosthodont 33, 25–34. doi: 10.1111/jopr.13784 37837403

[B10] Amaechi B. T. AbdulAzees P. A. Alshareif D. O. Shehata M. A. Lima P. Abdollahi A. . (2019a). Comparative efficacy of a hydroxyapatite and a fluoride toothpaste for prevention and remineralization of dental caries in children. BDJ Open 5, 18. doi: 10.1038/s41405-019-0026-8 31839988 PMC6901576

[B169] Amaechi B. T. Kasundra H. Okoye L. O. Tran P. L. Reid T. W. (2019b). Comparative efficacy in preventing plaque formation around pit and fissure sealants: a clinical trial. J. Contemp. Dent. Pract. 20, 531–536. doi: 10.5005/jp-journals-10024-2552 31316012

[B165] Amaechi B. T. McGarrell B. Luong M. N. Okoye L. O. Gakunga P. T. (2021). Prevention of white spot lesions around orthodontic brackets using organoselenium-containing antimicrobial enamel surface sealant. Heliyon. 7, e06490. doi: 10.1016/j.heliyon.2021.e06490 33748508 PMC7966844

[B166] Amaechi B. T. Najibfard K. Chedjieu I. P. Kasundra H. Okoye L. O. (2018). Do products preventing demineralization around orthodontic brackets affect adhesive bond strength? Open Dent. J. 12, 1029–1035. doi: 10.2174/1874210601812011029

[B9] Amaechi B. T. Tan A. I. Noureldin A. A. K. Kanthaiah K. Holladay E. Obiefuna A. C. . (2025). *In vitro* evaluation of the ability of nanohydroxyapatite toothpastes to enhance remineralization of enamel caries lesion. J. Dent. 161, 106006. doi: 10.1016/j.jdent.2025.106006 40744295

[B58] Apip C. Martínez A. Meléndrez M. Domínguez M. Marzialetti T. Báez R. . (2021). An *in vitro* study on the inhibition and ultrastructural alterations of Candida albicans biofilm by zinc oxide nanowires in a PMMA matrix. Saudi Dent. J. 33, 944–953. doi: 10.1016/j.sdentj.2021.08.006 34938036 PMC8665199

[B178] Armata Pharmaceuticals I (2020). Annual Report (Form 10-K) for Fiscal Year 2019. Annual Report (Form 10-K) (Marina del Rey, CA, USA: U.S. Securities and Exchange Commission).

[B101] Asadi Shahi S. Roudbar Mohammadi S. Roudbary M. Delavari H. (2019). A new formulation of graphene oxide/fluconazole compound as a promising agent against Candida albicans. Prog. Biomater. 8, 43–50. doi: 10.1007/s40204-019-0109-6 30859396 PMC6424987

[B63] Asahi R. Morikawa T. Ohwaki T. Aoki K. Taga Y. (2001). Visible-light photocatalysis in nitrogen-doped titanium oxides. Science 293, 269–271. doi: 10.1126/science.1061051 11452117

[B117] Bajunaid S. O. Baras B. H. Balhaddad A. A. Weir M. D. Xu H. H. K. (2021). Antibiofilm and protein-repellent polymethylmethacrylate denture base acrylic resin for treatment of denture stomatitis. Materials (Basel) 14, 1067. doi: 10.3390/ma14051067 33668779 PMC7956622

[B35] Barros J. Silva M. G. Rôças I. N. Gonçalves L. S. Alves F. F. Lopes M. A. . (2014a). Antibiofilm effects of endodontic sealers containing quaternary ammonium polyethylenimine nanoparticles. J. Endod. 40, 1167–1171. doi: 10.1016/j.joen.2013.12.021 25069926

[B34] Barros J. Silva M. G. Rodrigues M. A. Alves F. R. Lopes M. A. Pina-Vaz I. . (2014b). Antibacterial, physicochemical and mechanical properties of endodontic sealers containing quaternary ammonium polyethylenimine nanoparticles. Int. Endod. J. 47, 725–734. doi: 10.1111/iej.12213 24134748

[B30] Barszczewska-Rybarek I. M. Chrószcz M. W. Chladek G. (2021). Physicochemical and mechanical properties of Bis-GMA/TEGDMA dental composite resins enriched with quaternary ammonium polyethylenimine nanoparticles. Materials (Basel) 14, 2037. doi: 10.3390/ma14082037 33919544 PMC8074033

[B31] Beyth N. Pilo R. Weiss E. I. (2012). Antibacterial activity of dental cements containing quaternary ammonium polyethylenimine nanoparticles. J. Nanomater. 2012, 814763. doi: 10.1155/2012/814763

[B15] Beyth N. Yudovin-Farber I. Bahir R. Domb A. J. Weiss E. I. (2006). Antibacterial activity of dental composites containing quaternary ammonium polyethylenimine nanoparticles against *Streptococcus mutans*. Biomaterials 27, 3995–4002. doi: 10.1016/j.biomaterials.2006.03.003 16564083

[B29] Beyth N. Yudovin-Farber I. Perez-Davidi M. Domb A. J. Weiss E. I. (2010). Polyethyleneimine nanoparticles incorporated into resin composite cause cell death and trigger biofilm stress *in vivo*. Proc. Natl. Acad. Sci. U.S.A. 107, 22038–22043. doi: 10.1073/pnas.1010341107 21131569 PMC3009770

[B49] Bolenwar A. Reche A. Dhamdhere N. Rathi S. (2023). Applications of silver nanoparticles in dentistry. Cureus 15, e44090. doi: 10.7759/cureus.44090 37750112 PMC10518072

[B134] Caldeirão A. C. M. Araujo H. C. Tomasella C. M. Sampaio C. Dos Santos Oliveira M. J. Ramage G. . (2021). Effects of antifungal carriers based on chitosan-coated iron oxide nanoparticles on microcosm biofilms. Antibiotics (Basel Switzerland) 10, 588. doi: 10.3390/antibiotics10050588 34067527 PMC8155828

[B3] Centers for Disease Control and Prevention (2024). Oral Health Surveillance Report: Dental Caries, Tooth Retention, and Edentulism, United States, 2017-March 2020 (Atlanta (GA): Centers for Disease Control and Prevention).

[B163] Chaudiere J. Courtin O. Leclaire J. (1992). Glutathione oxidase activity of selenocystamine: a mechanistic study. Arch. Biochem. Biophys. 296, 328–336. doi: 10.1016/0003-9861(92)90580-p 1605642

[B90] Chen Y. W. Drury J. L. Chung W. O. Hobbs D. T. Wataha J. C. (2015). Titanates and titanate-metal compounds in biological contexts. Int. J. Med. Nano Res. 2, 9. doi: 10.23937/2378-3664/1410009 26430701 PMC4587770

[B43] Chen H. Gu L. Liao B. Zhou X. Cheng L. Ren B. (2020a). Advances of anti-caries nanomaterials. Molecules 25, 5047. doi: 10.3390/molecules25215047 33143140 PMC7662703

[B37] Chen H. Han Q. Zhou X. Zhang K. Wang S. Xu H. H. K. . (2017). Heat-polymerized resin containing dimethylaminododecyl methacrylate inhibits Candida albicans biofilm. Materials (Basel) 10, 431. doi: 10.3390/ma10040431 28772791 PMC5507000

[B149] Chen L. Jia L. Zhang Q. Zhou X. Liu Z. Li B. . (2017). A novel antimicrobial peptide against dental-caries-associated bacteria. Anaerobe 47, 165–172. doi: 10.1016/j.anaerobe.2017.05.016 28571698

[B96] Chen J. Zhao Q. Peng J. Yang X. Yu D. Zhao W. (2020). Antibacterial and mechanical properties of reduced graphene-silver nanoparticle nanocomposite modified glass ionomer cements. J. Dent. 96, 103332. doi: 10.1016/j.jdent.2020.103332 32283122

[B38] Chen H. Zhou Y. Zhou X. Liao B. Xu H. H. K. Chu C. H. . (2020b). Dimethylaminododecyl methacrylate inhibits Candida albicans and oropharyngeal candidiasis in a pH-dependent manner. Appl. Microbiol. Biotechnol. 104, 3585–3595. doi: 10.1007/s00253-020-10496-0 32125481

[B28] Chladek G. Barszczewska-Rybarek I. Chrószcz-Porębska M. Mertas A. (2023). The effect of quaternary ammonium polyethylenimine nanoparticles on bacterial adherence, cytotoxicity, and physical and mechanical properties of experimental dental composites. Sci. Rep. 13, 17497. doi: 10.1038/s41598-023-43851-y 37840040 PMC10577145

[B78] Chung W. O. Wataha J. C. Hobbs D. T. An J. Wong J. J. Park C. H. . (2011). Peroxotitanate- and monosodium metal-titanate compounds as inhibitors of bacterial growth. J. Biomed. Mater. Res. A. 97, 348–354. doi: 10.1002/jbm.a.33065 21472975

[B155] ClinicalTrials.gov . (2015). “ Study to evaluate the safety and microbiology of C16G2 in adolescent and adult dental subjects,” in Nct02509845. Bethesda, MD, USA: U.S. National Library of Medicine.

[B152] ClinicalTrials.gov (2022a). “ A study to evaluate the safety and microbiology of C16G2 varnish administered in multiple doses to dental subjects,” in Nct03004365. Bethesda, MD, USA: U.S. National Library of Medicine.

[B153] ClinicalTrials.gov (2022b). “ A study to evaluate the microbiology, safety and tolerability of C16G2 dental strip application,” in Nct03052842. Bethesda, MD, USA: U.S. National Library of Medicine.

[B154] ClinicalTrials.gov (2022c). “ A phase 2 study to evaluate the microbiology, safety and tolerability of C16G2 varnish and strip in adolescent and adult subjects,” in Nct03196219. Bethesda, MD, USA: U.S. National Library of Medicine.

[B44] Corrêa J. M. Mori M. Sanches H. L. da Cruz A. D. Poiate E. Jr. Poiate I. A. . (2015). Silver nanoparticles in dental biomaterials. Int. J. Biomater. 2015, 485275. doi: 10.1155/2015/485275 25667594 PMC4312639

[B70] da Cruz Nizer W. S. Adams M. E. Allison K. N. Montgomery M. C. Mosher H. Cassol E. . (2024). Oxidative stress responses in biofilms. Biofilm 7, 100203. doi: 10.1016/j.bioflm.2024.100203 38827632 PMC11139773

[B157] Dai L. L. Mei M. L. Chu C. H. Lo E. C. M. (2019). Mechanisms of bioactive glass on caries management: a review. Materials (Basel). 12, 4183. doi: 10.3390/ma12244183 31842454 PMC6947261

[B81] Deng S. Chung K. H. Chan D. Spiekerman C. (2016). Evaluation of bond strength and microleakage of a novel metal-titanate antibacterial agent. Oper. Dent. 41, E48–E56. doi: 10.2341/14-257-l 26652018

[B128] Dhall A. Self W. (2018). Cerium oxide nanoparticles: A brief review of their synthesis methods and biomedical applications. Antioxidants (Basel Switzerland) 7, 97. doi: 10.3390/antiox7080097 30042320 PMC6116044

[B56] do Amaral G. S. Negrini T. Maltz M. Arthur R. A. (2016). Restorative materials containing antimicrobial agents: is there evidence for their antimicrobial and anticaries effects? A systematic review. Aust. Dent. J. 61, 6–15. doi: 10.1111/adj.12338 26018839

[B125] Dong Q. Li Z. Xu J. Yuan Q. Chen L. Chen Z. (2022). Versatile graphitic nanozymes for magneto actuated cascade reaction-enhanced treatment of S. mutans biofilms. Nano Res. 15, 9800–9808. doi: 10.1007/s12274-022-4258-x30311153

[B16] Drejka P. Chrószcz-Porębska M. Kazek-Kęsik A. Chladek G. Barszczewska-Rybarek I. (2024). Chemical modification of dental dimethacrylate copolymer with tetramethylxylylene diisocyanate-based quaternary ammonium urethane-dimethacrylates-physicochemical, mechanical, and antibacterial properties. Materials (Basel) 17, 298. doi: 10.3390/ma17020298 38255466 PMC10817292

[B137] Eckert R. He J. Yarbrough D. K. Qi F. Anderson M. H. Shi W. (2006a). Targeted killing of *Streptococcus mutans* by a pheromone-guided "smart" antimicrobial peptide. Antimicrob. Agents Chemother. 50, 3651–3657. doi: 10.1128/AAC.00622-06 PMC1635210 17060534

[B136] Eckert R. Qi F. Yarbrough D. K. He J. Anderson M. H. Shi W. (2006b). Adding selectivity to antimicrobial peptides: Rational design of a multidomain peptide against Pseudomonas spp. Antimicrob. Agents Chemother. 50, 1480–1488. doi: 10.1128/aac.50.4.1480-1488.2006 16569868 PMC1426969

[B138] Eckert R. Sullivan R. Shi W. (2012). Targeted antimicrobial treatment to re-establish a healthy microbial flora for long-term protection. Adv. Dent. Res. 24, 94–97. doi: 10.1177/0022034512453725 22899688 PMC3420366

[B79] Eiampongpaiboon T. Chung W. O. Bryers J. D. Chung K. H. Chan D. C. N. (2015). Antibacterial activity of gold-titanates on gram-positive cariogenic bacteria. Acta Biomater. Odontol. Scand. 1, 51–58. doi: 10.3109/23337931.2015.1084883 28642901 PMC5433202

[B45] Emram R. Sionov R. V. Gutkin V. Wilensky A. Steinberg D. Assad R. (2025). Mechanism of action of zinc oxide nanoparticles as an antibacterial agent against *Streptococcus mutans*. Biomolecules 15, 1660. doi: 10.3390/biom15121660 41463315 PMC12730517

[B74] Esteban Florez F. L. Hiers R. D. Larson P. Johnson M. O'Rear E. Rondinone A. J. . (2018). Antibacterial dental adhesive resins containing nitrogen-doped titanium dioxide nanoparticles. Mater. Sci. Eng. C. Mater. Biol. Appl. 93, 931–943. doi: 10.1016/j.msec.2018.08.060 30274130 PMC6500090

[B76] Esteban Florez F. L. Kraemer H. Hiers R. D. Sacramento C. M. Rondinone A. J. Silvério K. G. . (2020a). Sorption, solubility and cytotoxicity of novel antibacterial nanofilled dental adhesive resins. Sci. Rep. 10, 13503. doi: 10.1038/s41598-020-70487-z 32782299 PMC7421579

[B75] Esteban Florez F. L. Trofimov A. A. Ievlev A. Qian S. Rondinone A. J. Khajotia S. S. (2020b). Advanced characterization of surface-modified nanoparticles and nanofilled antibacterial dental adhesive resins. Sci. Rep. 10, 9811. doi: 10.1038/s41598-020-66819-8 32555360 PMC7299952

[B11] Falsetta M. L. Klein M. I. Colonne P. M. Scott-Anne K. Gregoire S. Pai C. H. . (2014). Symbiotic relationship between *Streptococcus mutans* and Candida albicans synergizes virulence of plaque biofilms *in vivo*. Infect. Immun. 82, 1968–1981. doi: 10.1128/IAI.00087-14 24566629 PMC3993459

[B84] Fatani E. J. Almutairi H. H. Alharbi A. O. Alnakhli Y. O. Divakar D. D. Muzaheed . (2017). *In vitro* assessment of stainless steel orthodontic brackets coated with titanium oxide mixed Ag for anti-adherent and antibacterial properties against *Streptococcus mutans* and Porphyromonas gingivalis. Microb. Pathog. 112, 190–194. doi: 10.1016/j.micpath.2017.09.052 28966064

[B4] Featherstone J. D. B. (2022). Dental restorative materials containing quaternary ammonium compounds have sustained antibacterial action. J. Am. Dent. Assoc. 153, 1114–1120. doi: 10.1016/j.adaj.2022.09.006 36272816

[B86] Felix Gomez G. G. Lippert F. Ando M. Zandona A. F. Eckert G. J. Gregory R. L. (2018). Effect of violet-blue light on *Streptococcus mutans*-induced enamel demineralization. Dent. J. (Basel) 6, 6. doi: 10.3390/dj6020006 29565266 PMC6023538

[B87] Felix Gomez G. G. Lippert F. Ando M. Zandona A. F. Eckert G. J. Gregory R. L. (2019). Photoinhibition of *Streptococcus mutans* biofilm-induced lesions in human dentin by violet-blue light. Dent. J. (Basel) 7, 113. doi: 10.3390/dj7040113 31835833 PMC6960986

[B146] Fu Y. Cannon R. D. Li K. C. Ekambaram M. Cooper P. R. Mei M. L. (2025). Development and characterisation of a novel antimicrobial peptide GA-C16G2 targeting *Streptococcus mutans*. J. Dent. 161, 105927. doi: 10.1016/j.jdent.2025.105927 40578786

[B116] Fujiwara N. Yumoto H. Miyamoto K. Hirota K. Nakae H. Tanaka S. . (2019). 2-methacryloyloxyethyl phosphorylcholine (MPC)-polymer suppresses an increase of oral bacteria: A single-blind, crossover clinical trial. Clin. Oral. Investig. 23, 739–746. doi: 10.1007/s00784-018-2490-2 29766378 PMC7736026

[B122] Gao L. Liu Y. Kim D. Li Y. Hwang G. Naha P. C. . (2016). Nanocatalysts promote *Streptococcus mutans* biofilm matrix degradation and enhance bacterial killing to suppress dental caries *in vivo*. Biomaterials 101, 272–284. doi: 10.1016/j.biomaterials.2016.05.051 27294544 PMC4949957

[B39] Gao B. Zhang X. Zhu Y. (2007). Studies on the preparation and antibacterial properties of quaternized polyethyleneimine. J. Biomater. Sci. Polym. Ed. 18, 531–544. doi: 10.1163/156856207780852523 17550657

[B2] GBD 2021 Oral Disorders Collaborators (2025). Trends in the global, regional, and national burden of oral conditions from 1990 to 2021: a systematic analysis for the Global Burden of Disease Study 2021. Lancet 405, 897–910. doi: 10.1016/S0140-6736(24)02811-3 40024264

[B133] Ghaffari M. Nilsson M. Tolker-Nielsen T. Taheri-Kafrani A. (2026). Peroxidase-mimicking chitosan/dextran-coated cobalt ferrite-graphene oxide nanozyme enhances hydrogen peroxide-mediated killing of cariogenic pathogens in dental biofilm models. Sci. Rep. 16, 17484. doi: 10.1038/s41598-026-43315-z 41981039 PMC13237087

[B106] Gholami A. Emadi F. Amini A. Shokripour M. Chashmpoosh M. Omidifar N. (2020). Functionalization of graphene oxide nanosheets can reduce their cytotoxicity to dental pulp stem cells. J. Nanomater. 2020, 6942707. doi: 10.1155/2020/6942707

[B172] Gong T. Tang B. Zhou X. Zeng J. Lu M. Guo X. . (2018). Genome editing in *Streptococcus mutans* through self-targeting CRISPR arrays. Mol. Oral. Microbiol. 33, 440–449. doi: 10.1111/omi.12247 30329221

[B127] Guo J. Liu M. D. Lei W. Xu Y. Li K. Yu J. . (2023). Ultrasound activatable microbombs via co-loading CaO2/perfluorohexane for cascade catalytic biofilm disruption and caries prevention. Adv. Funct. Mater. 33, 2213729. doi: 10.1002/adfm.20221372942587375

[B64] Gupta K. Singh R. P. Pandey A. Pandey A. (2013). Photocatalytic antibacterial performance of TiO2 and Ag-doped TiO2 against S. aureus. P. aeruginosa and E. coli. Beilstein J. Nanotechnol. 4, 345–351. doi: 10.3762/bjnano.4.40 PMC3701421 23844339

[B160] He Y. Vasilev K. Zilm P. (2023). pH-responsive biomaterials for the treatment of dental caries-a focussed and critical review. Pharmaceutics. 15, 1837. doi: 10.3390/pharmaceutics15071837 37514024 PMC10385394

[B140] He J. Yarbrough D. K. Kreth J. Anderson M. H. Shi W. Eckert R. (2010). Systematic approach to optimizing specifically targeted antimicrobial peptides against *Streptococcus mutans*. Antimicrob. Agents Chemother. 54, 2143–2151. doi: 10.1128/aac.01391-09 20211885 PMC2863653

[B92] He J. Zhu X. Qi Z. Wang C. Mao X. Zhu C. . (2015). Killing dental pathogens using antibacterial graphene oxide. ACS Appl. Mater. Interfaces 7, 5605–5611. doi: 10.1021/acsami.5b01069 25705785

[B77] Hiers R. D. Huebner P. Khajotia S. S. Florez F. L. E. (2022). Characterization of experimental nanoparticulated dental adhesive resins with long-term antibacterial properties. Nanomaterials (Basel) 12, 3732. doi: 10.3390/nano12213732 36364508 PMC9656602

[B161] Horev B. Klein M. I. Hwang G. Li Y. Kim D. Koo H. . (2015). pH-activated nanoparticles for controlled topical delivery of farnesol to disrupt oral biofilm virulence. ACS Nano 9, 2390–2404. doi: 10.1021/nn507170s 25661192 PMC4395463

[B89] Huang X. Darveau R. McLean J. Chan D. (2022). “ Gold titanate modulates the virulence of cariogenic oral biofilms,” in 2022 Aadocr/Cadr Annual Meeting. Alexandria, VA, USA: American Association for Dental, Oral, and Craniofacial Research (AADOCR).

[B131] Huang Y. Liu Y. Pandey N. K. Shah S. Simon-Soro A. Hsu J. C. . (2023). Iron oxide nanozymes stabilize stannous fluoride for targeted biofilm killing and synergistic oral disease prevention. Nat. Commun. 14, 6087. doi: 10.1038/s41467-023-41687-8 37773239 PMC10541875

[B124] Huang Y. Liu Y. Shah S. Kim D. Simon-Soro A. Ito T. . (2021). Precision targeting of bacterial pathogen via bi-functional nanozyme activated by biofilm microenvironment. Biomaterials 268, 120581. doi: 10.1016/j.biomaterials.2020.120581 33302119 PMC8182968

[B1] Huang X. Zandoná A. F. (2025). Novel strategies for caries control. Front. Cell. Infect. Microbiol. 15, 1756613. doi: 10.3389/fcimb.2025.1756613 41488480 PMC12757386

[B7] Hussain U. Wahab A. Kamran M. A. Alnazeh A. A. Almoammar S. Alshahrani S. S. M. . (2025). Prevalence, incidence and risk factors of white spot lesions associated with orthodontic treatment - a systematic review and meta-analysis. Orthod. Craniofac Res. 28, 379–399. doi: 10.1111/ocr.12888 39717964

[B115] Ikeya K. Iwasa F. Inoue Y. Fukunishi M. Takahashi N. Ishihara K. . (2018). Inhibition of denture plaque deposition on complete dentures by 2-methacryloyloxyethyl phosphorylcholine polymer coating: A clinical study. J. Prosthet. Dent. 119, 67–74. doi: 10.1016/j.prosdent.2017.02.012 28506653

[B18] Imazato S. (2003). Antibacterial properties of resin composites and dentin bonding systems. Dent. Mater. 19, 449–457. doi: 10.1016/S0109-5641(02)00102-1 12837391

[B14] Imazato S. Torii M. Tsuchitani Y. McCabe J. F. Russell R. R. (1994). Incorporation of bacterial inhibitor into resin composite. J. Dent. Res. 73, 1437–1443. doi: 10.1177/00220345940730080701 8083440

[B179] International Organization for Standardization (2023). ISO 3990:2023 Dentistry—Evaluation of Antibacterial Activity of Dental Restorative Materials, Luting Materials, Fissure Sealants and Orthodontic Bonding or Luting Materials (Geneva, Switzerland: International Organization for Standardization).

[B150] Jiang W. Liu J. Chen A. Zhao T. Xu Z. Xu L. . (2026). Antimicrobial peptides as novel ecological approaches to caries prevention. Drug Des. Devel Ther. 20, 592732. doi: 10.2147/DDDT.S592732 41918966 PMC13034794

[B145] Jiang W. Wang Y. Luo J. Chen X. Zeng Y. Li X. . (2020). Antimicrobial peptide GH12 prevents dental caries by regulating dental plaque microbiota. Appl. Environ. Microbiol. 86, e00527-20. doi: 10.1128/AEM.00527-20 32414800 PMC7357485

[B144] Jiang W. Wang Y. Luo J. Li X. Zhou X. Li W. . (2018). Effects of antimicrobial peptide GH12 on the cariogenic properties and composition of a cariogenic multispecies biofilm. Appl. Environ. Microbiol. 84, e01423-18. doi: 10.1128/AEM.01423-18 30341079 PMC6275336

[B139] Kaplan C. W. Sim J. H. Shah K. R. Kolesnikova-Kaplan A. Shi W. Eckert R. (2011). Selective membrane disruption: Mode of action of C16G2, a specifically targeted antimicrobial peptide. Antimicrob. Agents Chemother. 55, 3446–3452. doi: 10.1128/aac.00342-11 21518845 PMC3122425

[B50] Kasraei S. Sami L. Hendi S. Alikhani M. Y. Rezaei-Soufi L. Khamverdi Z. (2014). Antibacterial properties of composite resins incorporating silver and zinc oxide nanoparticles on *Streptococcus mutans* and Lactobacillus. Restor. Dent. Endod. 39, 109–114. doi: 10.5395/rde.2014.39.2.109 PMC3978100 24790923

[B33] Kesler Shvero D. Abramovitz I. Zaltsman N. Perez Davidi M. Weiss E. I. Beyth N. (2013). Towards antibacterial endodontic sealers using quaternary ammonium nanoparticles. Int. Endod. J. 46, 747–754. doi: 10.1111/iej.12054 23402669

[B173] Kiga K. Tan X. E. Ibarra-Chávez R. Watanabe S. Aiba Y. Sato’o Y. . (2020). Development of CRISPR-Cas13a-based antimicrobials capable of sequence-specific killing of target bacteria. Nat. Commun. 11, 2934. doi: 10.1038/s41467-020-16731-6 32523110 PMC7287087

[B5] Kopperud S. E. Tveit A. B. Gaarden T. Sandvik L. Espelid I. (2012). Longevity of posterior dental restorations and reasons for failure. Eur. J. Oral. Sci. 120, 539–548. doi: 10.1111/eos.12004 23167471

[B164] Kramer G. F. Ames B. N. (1988). Mechanisms of mutagenicity and toxicity of sodium selenite (Na2SeO3) in Salmonella typhimurium. Mutat. Res. Fundam. Mol. Mech. Mutagen. 201, 169–180. doi: 10.1016/0027-5107(88)90123-6 2843765

[B95] Kulshrestha S. Khan S. Meena R. Singh B. R. Khan A. U. (2014). A graphene/zinc oxide nanocomposite film protects dental implant surfaces against cariogenic *Streptococcus mutans*. Biofouling 30, 1281–1294. doi: 10.1080/08927014.2014.983093 25431994

[B114] Kwon J. S. Kim J. Y. Mangal U. Seo J. Y. Lee M. J. Jin J. . (2021). Durable oral biofilm resistance of 3D-printed dental base polymers containing zwitterionic materials. Int. J. Mol. Sci. 22, 417. doi: 10.3390/ijms22010417 33401545 PMC7795277

[B112] Lee M. J. Mangal U. Kim S. J. Yoon Y. P. Ahn E. S. Jang E. S. . (2020). Improvement in the microbial resistance of resin-based dental sealant by sulfobetaine methacrylate incorporation. Polymers (Basel) 12, 1716. doi: 10.3390/polym12081716 32751729 PMC7463993

[B141] Li L. N. Guo L. H. Lux R. Eckert R. Yarbrough D. He J. . (2010). Targeted antimicrobial therapy against *Streptococcus mutans* establishes protective non-cariogenic oral biofilms and reduces subsequent infection. Int. J. Oral. Sci. 2, 66–73. doi: 10.4248/ijos10024 20737932 PMC3733586

[B42] Li F. Weir M. D. Fouad A. F. Xu H. H. (2014). Effect of salivary pellicle on antibacterial activity of novel antibacterial dental adhesives using a dental plaque microcosm biofilm model. Dent. Mater. 30, 182–191. doi: 10.1016/j.dental.2013.11.004 24332270 PMC4023513

[B20] Li F. Weir M. D. Xu H. H. (2013). Effects of quaternary ammonium chain length on antibacterial bonding agents. J. Dent. Res. 92, 932–938. doi: 10.1177/0022034513502053 23958761 PMC3775374

[B132] Liao J. Zhang L. Sun B. Wang D. Zhang Z. Ma W. . (2024). Stimuli-responsive graphdiyne-silver nanozymes for catalytic ion therapy of dental caries through targeted biofilms removal and remineralization. Nano Today 55, 102204. doi: 10.1016/j.nantod.2024.10220442574925

[B130] Liu Y. Huang Y. Kim D. Ren Z. Oh M. J. Cormode D. P. . (2021). Ferumoxytol nanoparticles target biofilms causing tooth decay in the human mouth. Nano Lett. 21, 9442–9449. doi: 10.1021/acs.nanolett.1c02702 34694125 PMC9308480

[B129] Liu Y. Naha P. C. Hwang G. Kim D. Huang Y. Simon-Soro A. . (2018). Topical ferumoxytol nanoparticles disrupt biofilms and prevent tooth decay *in vivo* via intrinsic catalytic activity. Nat. Commun. 9, 2920. doi: 10.1038/s41467-018-05342-x 30065293 PMC6068184

[B94] Liu S. Zeng T. H. Hofmann M. Burcombe E. Wei J. Jiang R. . (2011). Antibacterial activity of graphite, graphite oxide, graphene oxide, and reduced graphene oxide: membrane and oxidative stress. ACS Nano 5, 6971–6980. doi: 10.1021/nn202451x 21851105

[B121] Liu L. Zhang Y. Ju T. Chen X. Li X. Wu L. A. (2024). Nanozymes: A promising solution for dental antibacterial applications. RSC Adv. 14, 36945–36959. doi: 10.1039/D4RA07303G 39569116 PMC11577344

[B147] Liu Q. Zhou L. Peng S. Li Q. L. Wong H. M. (2025). Antimicrobial peptide P-113-DPS suppresses the cariogenic virulence of *Streptococcus mutans*. ACS Appl. Bio Mater. 8, 4973–4980. doi: 10.1021/acsabm.5c00314 40452139 PMC12175158

[B48] Ma X. Zhou S. Xu X. Du Q. (2022). Copper-containing nanoparticles: Mechanism of antimicrobial effect and application in dentistry-a narrative review. Front. Surg. 9, 905892. doi: 10.3389/fsurg.2022.905892 35990090 PMC9388913

[B88] Mančić L. Almeida L. A. Machado T. M. Gil-Londoño J. Dinić I. Tomić M. . (2023). Tetracycline removal through the synergy of catalysis and photocatalysis by novel NaYF(4):Yb,Tm@TiO(2)-acetylacetone hybrid core-shell structures. Int. J. Mol. Sci. 24, 9441. doi: 10.3390/ijms24119441 PMC10253680 37298390

[B107] Mangal U. Kwon J. S. Choi S. H. (2020). Bio-interactive zwitterionic dental biomaterials for improving biofilm resistance: Characteristics and applications. Int. J. Mol. Sci. 21, 9087. doi: 10.3390/ijms21239087 33260367 PMC7730019

[B98] Mao M. Zhang W. Huang Z. Huang J. Wang J. Li W. . (2021). Graphene oxide-copper nanocomposites suppress cariogenic *Streptococcus mutans* biofilm formation. Int. J. Nanomedicine 16, 7727–7739. doi: 10.2147/ijn.s303521 34824531 PMC8610231

[B102] Martuci R. Oliveira S. J. Martuci M. Reis-Campos J. Figueiral M. H. (2025). Antimicrobial effect of graphene in dentistry: A scoping review. Dent. J. (Basel) 13, 355. doi: 10.3390/dj13080355 40863058 PMC12384847

[B119] Masotti E. Poma N. Guazzelli E. Fiaschi I. Glisenti A. Vivaldi F. . (2020). Fluorinated vs. zwitterionic-polymer grafted surfaces for adhesion prevention of the fungal pathogen Candida albicans. Polymers (Basel) 12, 398. doi: 10.3390/polym12020398 32050664 PMC7077729

[B8] Melo M. A. S. Garcia I. M. Mokeem L. Weir M. D. Xu H. H. K. Montoya C. . (2023). Developing bioactive dental resins for restorative dentistry. J. Dent. Res. 102, 1180–1190. doi: 10.1177/00220345231182357 37555431 PMC11066520

[B62] Mohammadi H. Moradpoor H. Beddu S. Mozaffari H. R. Sharifi R. Rezaei R. . (2025). Current trends and research advances on the application of TiO2 nanoparticles in dentistry: how far are we from clinical translation? Heliyon 11, e42169. doi: 10.1016/j.heliyon.2025.e42169 39991247 PMC11847115

[B82] Monica A. Padmanabhan S. (2022). The effect of nitrogen-doped titanium dioxide-modified stainless steel brackets on *Streptococcus mutans*. Angle Orthod. 92, 396–401. doi: 10.2319/062521-510.1 35072710 PMC9020397

[B13] Montoya C. Roldan L. Yu M. Valliani S. Ta C. Yang M. . (2023). Smart dental materials for antimicrobial applications. Bioact. Mater. 24, 1–19. doi: 10.1016/j.bioactmat.2022.12.002 36582351 PMC9763696

[B67] Moradpoor H. Safaei M. Golshah A. Mozaffari H. R. Sharifi R. Imani M. M. . (2021). Green synthesis and antifungal effect of titanium dioxide nanoparticles on oral Candida albicans pathogen. Inorg. Chem. Commun. 130, 108748. doi: 10.1016/j.inoche.2021.10874842574925

[B104] Moreira E. M. R. U. Abreu M. A. P. Frazão M. C. A. Ferreira P. V. C. Bauer J. Carvalho C. N. . (2024). Effect of graphene oxide nanoparticles incorporation on the mechanical properties of a resin-modified glass ionomer cement. Polymers (Basel) 16, 2401. doi: 10.3390/polym16172401 39274034 PMC11396858

[B59] Mousavi S. A. Ghotaslou R. Khorramdel A. Akbarzadeh A. Aeinfar A. (2020). Antibacterial and antifungal impacts of combined silver, zinc oxide, and chitosan nanoparticles within tissue conditioners of complete dentures *in vitro*. Ir. J. Med. Sci. 189, 1343–1350. doi: 10.1007/s11845-020-02243-1 32405923

[B54] Nagireddy V. R. Reddy D. Kondamadugu S. Puppala N. Mareddy A. Chris A. (2019). Nanosilver fluoride-a paradigm shift for arrest in dental caries in primary teeth of schoolchildren: a randomized controlled clinical trial. Int. J. Clin. Pediatr. Dent. 12, 484–490. doi: 10.5005/jp-journals-10005-1703 32440060 PMC7229382

[B123] Naha P. C. Liu Y. Hwang G. Huang Y. Gubara S. Jonnakuti V. . (2019). Dextran-coated iron oxide nanoparticles as biomimetic catalysts for localized and pH-activated biofilm disruption. ACS Nano 13, 4960–4971. doi: 10.1021/acsnano.8b08702 30642159 PMC7059368

[B156] Namburu J. R. Rajendra Sanosh A. B. Poosarla C. S. Manthapuri S. Pinnaka M. Baddam V. R. R. (2022). *Streptococcus mutans*-specific antimicrobial peptide C16G2-mediated caries prevention: a review. Front. Dent. 19, 17. doi: 10.18502/fid.v19i17.9963 36458273 PMC9675619

[B91] Nizami M. Z. I. Yin I. X. Lung C. Y. K. Niu J. Y. Mei M. L. Chu C. H. (2022). *In vitro* studies of graphene for management of dental caries and periodontal disease: a concise review. Pharmaceutics 14, 1997. doi: 10.3390/pharmaceutics14101997 36297434 PMC9611330

[B27] Pietrokovski Y. Nisimov I. Kesler-Shvero D. Zaltsman N. Beyth N. (2016). Antibacterial effect of composite resin foundation material incorporating quaternary ammonium polyethyleneimine nanoparticles. J. Prosthet. Dent. 116, 603–609. doi: 10.1016/j.prosdent.2016.02.022 27157602

[B73] Poosti M. Ramazanzadeh B. Zebarjad M. Javadzadeh P. Naderinasab M. Shakeri M. T. (2013). Shear bond strength and antibacterial effects of orthodontic composite containing TiO2 nanoparticles. Eur. J. Orthod. 35, 676–679. doi: 10.1093/ejo/cjs073 23264617

[B52] Porter G. C. Tompkins G. R. Schwass D. R. Li K. C. Waddell J. N. Meledandri C. J. (2020). Anti-biofilm activity of silver nanoparticle-containing glass ionomer cements. Dent. Mater. 36, 1096–1107. doi: 10.1016/j.dental.2020.05.001 32505537

[B6] Preshaw P. M. Walls A. W. Jakubovics N. S. Moynihan P. J. Jepson N. J. Loewy Z. (2011). Association of removable partial denture use with oral and systemic health. J. Dent. 39, 711–719. doi: 10.1016/j.jdent.2011.08.018 21924317

[B176] Pulingam T. Thong K. L. Ali M. E. Appaturi J. N. Dinshaw I. J. Ong Z. Y. . (2019). Graphene oxide exhibits differential mechanistic action towards Gram-positive and Gram-negative bacteria. Colloids Surf. B. Biointerfaces. 181, 6–15. doi: 10.1016/j.colsurfb.2019.05.023 31103799

[B118] Putra Wigianto A. Y. Ishida Y. Iwawaki Y. Goto T. Watanabe M. Sekine K. . (2023). 2-methacryloyloxyethyl phosphorylcholine polymer treatment prevents Candida albicans biofilm formation on acrylic resin. J. Prosthodont Res. 67, 384–391. doi: 10.2186/jpr.JPR_D_22_00102 36288959

[B61] Qi J. Liu H. Li H. Liu H. Wang Y. Kong C. . (2025). How TiO2 nanomaterials are emerging as key therapeutics in stomatology. Int. J. Nanomedicine 20, 11451–11478. doi: 10.2147/IJN.S533650 40995016 PMC12456319

[B55] Quritum M. Abdella A. Amer H. El Desouky L. M. El Tantawi M. (2024). Effectiveness of nanosilver fluoride and silver diamine fluoride in arresting early childhood caries: a randomized controlled clinical trial. BMC Oral. Health 24, 701. doi: 10.1186/s12903-024-04406-3 38890627 PMC11184862

[B12] Ramburrun P. Pringle N. A. Dube A. Adam R. Z. D'Souza S. Aucamp M. (2021). Recent advances in the development of antimicrobial and antifouling biocompatible materials for dental applications. Materials (Basel) 14, 3167. doi: 10.3390/ma14123167 34207552 PMC8229368

[B174] Saffari Natanzi A. Poudineh M. Karimi E. Khaledi A. Haddad Kashani H. (2025). Innovative approaches to combat antibiotic resistance: integrating CRISPR/Cas9 and nanoparticles against biofilm-driven infections. BMC Med. 23, 486. doi: 10.1186/s12916-025-04323-4 40830872 PMC12366227

[B83] Salehi P. Babanouri N. Roein-Peikar M. Zare F. (2018). Long-term antimicrobial assessment of orthodontic brackets coated with nitrogen-doped titanium dioxide against *Streptococcus mutans*. Prog. Orthod. 19, 35. doi: 10.1186/s40510-018-0236-y 30221309 PMC6139290

[B162] Seko Y. Imura N. (1997). Active oxygen generation as a possible mechanism of selenium toxicity. Biomed. Environ. Sci. 10, 333–339. 9315327

[B47] Sharifian S. Loghmani A. Nayyerain S. Javanbakht S. Daneii P. (2023). Application of magnesium oxide nanoparticles in dentistry: a literature review. Eur. J. Gen. Dent. 12, 1–6. doi: 10.1055/s-0042-17606733785596

[B36] Sharon E. Sharabi R. Eden A. Zabrovsky A. Ben-Gal G. Sharon E. . (2018). Antibacterial activity of orthodontic cement containing quaternary ammonium polyethylenimine nanoparticles adjacent to orthodontic brackets. Int. J. Environ. Res. Public Health 15, 606. doi: 10.3390/ijerph15040606 29584643 PMC5923648

[B21] Shvero D. K. Zatlsman N. Hazan R. Weiss E. I. Beyth N. (2015). Characterisation of the antibacterial effect of polyethyleneimine nanoparticles in relation to particle distribution in resin composite. J. Dent. 43, 287–294. doi: 10.1016/j.jdent.2014.05.003 24881908

[B159] Skallevold H. E. Rokaya D. Khurshid Z. Zafar M. S. (2019). Bioactive glass applications in dentistry. Int. J. Mol. Sci. 20, 5960. doi: 10.3390/ijms20235960 31783484 PMC6928922

[B46] Sodagar A. Akhoundi M. S. A. Bahador A. Jalali Y. F. Behzadi Z. Elhaminejad F. . (2017). Effect of TiO2 nanoparticles incorporation on antibacterial properties and shear bond strength of dental composite used in orthodontics. Dental Press J. Orthod. 22, 67–74. doi: 10.1590/2177-6709.22.5.067-074.oar 29160346 PMC5730138

[B65] Solanki L. A. Dinesh S. P. S. Jain R. K. Balasubramaniam A. (2023). Effects of titanium oxide coating on the antimicrobial properties, surface characteristics, and cytotoxicity of orthodontic brackets - a systematic review and meta analysis of in-vitro studies. J. Oral. Biol. Craniofac Res. 13, 553–562. doi: 10.1016/j.jobcr.2023.05.014 37409325 PMC10319217

[B57] Subramaniam J. Varghese R. M. Subramanian A. K. Shanmugam R. (2024). Mechanism of action of green-synthesized silver nanoparticle-incorporated dental varnish against Candida albicans. Cureus 16, e69353. doi: 10.7759/cureus.69353 39398763 PMC11471302

[B151] Sullivan R. Santarpia P. Lavender S. Gittins E. Liu Z. Anderson M. H. . (2011). Clinical efficacy of a specifically targeted antimicrobial peptide mouth rinse: targeted elimination of *Streptococcus mutans* and prevention of demineralization. Caries Res. 45, 415–428. doi: 10.1159/000330510 21860239 PMC3169368

[B111] Sun F. Hung H. C. Yan W. Wu K. Shimchuk A. A. Gray S. D. . (2021). Inhibition of oral biofilm formation by zwitterionic nonfouling coating. J. Biomed. Mater. Res. B. Appl. Biomater. 109, 1418–1425. doi: 10.1002/jbm.b.34801 33538114

[B41] Szymczak-Pajor I. Zalega M. Nowak J. Śliwińska A. Woźniak K. Bociong K. (2025). Cytotoxicity and genotoxicity of resin-based dental composites modified with quaternary ammonium salts. J. Funct. Biomater. 16, 459. doi: 10.3390/jfb16120459 41440636 PMC12734297

[B60] Tabari K. Hosseinpour S. Parashos P. Kardouni Khozestani P. Rahimi H. M. (2017). Cytotoxicity of selected nanoparticles on human dental pulp stem cells. Iran. Endod. J. 12, 137–142. doi: 10.22037/iej.2017.28 PMC5421267 28496518

[B103] Teimoorian M. Mirzaie M. Tashakkorian H. Gholinia H. Alaghemand H. Pournajaf A. . (2023). Effects of adding functionalized graphene oxide nanosheets on physical, mechanical, and anti-biofilm properties of acrylic resin: *In vitro*- experimental study. Dent. Res. J. (Isfahan) 20, 37. doi: 10.4103/1735-3327.372654 PMC10166752 37180695

[B80] Theda L. Kim P. Chan D. C. N. Chung W. O. (2018). Titanate-metal complex as a novel antimicrobial in dentistry. Dental Oral. Biol. Craniofacial Res. 1(2), 4–6. doi: 10.31487/j.DOBCR.2018.02.003

[B19] Tiller J. C. Liao C. J. Lewis K. Klibanov A. M. (2001). Designing surfaces that kill bacteria on contact. Proc. Natl. Acad. Sci. U.S.A. 98, 5981–5985. doi: 10.1073/pnas.111143098 11353851 PMC33409

[B110] Torres L. Jr Bienek D. R. (2020). Use of protein repellents to enhance the antimicrobial functionality of quaternary ammonium containing dental materials. J. Funct. Biomater. 11, 54. doi: 10.3390/jfb11030054 32752169 PMC7565790

[B170] Tran P. Hamood A. Mosley T. Gray T. Jarvis C. Webster D. . (2013). Organo-selenium-containing dental sealant inhibits bacterial biofilm. J. Dent. Res. 92, 461–466. doi: 10.1177/0022034513482141 23475900 PMC3627506

[B171] Tran P. L. Patel S. Hamood A. N. Enos T. Mosley T. Jarvis C. . (2014). A novel organo-selenium bandage that inhibits biofilm development in a wound by gram-positive and gram-negative wound pathogens. Antibiotics. 3, 435–449. doi: 10.3390/antibiotics3030435 27025754 PMC4790367

[B85] Venkatesan K. Kailasam V. Padmanabhan S. (2020). Evaluation of titanium dioxide coating on surface roughness of nickel-titanium archwires and its influence on *Streptococcus mutans* adhesion and enamel mineralization: a prospective clinical study. Am. J. Orthod. Dentofacial Orthop. 158, 199–208. doi: 10.1016/j.ajodo.2019.07.019 32576426

[B158] Waltimo T. Brunner T. J. Vollenweider M. Stark W. J. Zehnder M. (2007). Antimicrobial effect of nanometric bioactive glass 45S5. J. Dent. Res. 86, 754–757. doi: 10.1177/154405910708600813 17652205

[B143] Wang Y. Fan Y. Zhou Z. Tu H. Ren Q. Wang X. . (2017). De novo synthetic short antimicrobial peptides against cariogenic bacteria. Arch. Oral. Biol. 80, 41–50. doi: 10.1016/j.archoralbio.2017.03.017 28366785

[B51] Wang J. Jiang W. Liang J. Ran S. (2022). Influence of silver nanoparticles on the resin-dentin bond strength and antibacterial activity of a self-etch adhesive system. J. Prosthet. Dent. 128, 1363.e1–.e10. doi: 10.1016/j.prosdent.2022.09.015 36396489

[B105] Wang K. Ruan J. Song H. Zhang J. Wo Y. Guo S. . (2011). Biocompatibility of graphene oxide. Nanoscale Res. Lett. 6, 8. doi: 10.1007/s11671-010-9751-6 27502632 PMC3212228

[B126] Wang Y. Shen X. Ma S. Guo Q. Zhang W. Cheng L. . (2020). Oral biofilm elimination by combining iron-based nanozymes and hydrogen peroxide-producing bacteria. Biomater. Sci. 8, 2447–2458. doi: 10.1039/c9bm01889a 32096497

[B24] Wang H. Wang S. Cheng L. Jiang Y. Melo M. A. S. Weir M. D. . (2019). Novel dental composite with capability to suppress cariogenic species and promote non-cariogenic species in oral biofilms. Mater. Sci. Eng. C. Mater. Biol. Appl. 94, 587–596. doi: 10.1016/j.msec.2018.10.004 30423744 PMC6239200

[B142] Wang Y. Wang X. Jiang W. Wang K. Luo J. Li W. . (2018). Antimicrobial peptide GH12 suppresses cariogenic virulence factors of *Streptococcus mutans*. J. Oral. Microbiol. 10, 1442089. doi: 10.1080/20002297.2018.1442089 29503706 PMC5827641

[B23] Wang S. Wang H. Ren B. Li X. Wang L. Zhou H. . (2018). Drug resistance of oral bacteria to new antibacterial dental monomer dimethylaminohexadecyl methacrylate. Sci. Rep. 8, 5509. doi: 10.1038/s41598-018-23831-3 29615732 PMC5882658

[B53] Wassel M. O. Allam G. G. (2022). Anti-Bacterial effect, fluoride release, and compressive strength of a glass ionomer containing silver and titanium nanoparticles. Indian J. Dent. Res. 33, 75–79. doi: 10.4103/ijdr.ijdr_117_20 35946249

[B148] Wei J. Chen Z. Li S. Zhou S. Riaz A. Ma S. . (2026). A potent dual-targeting antimicrobial peptide specifically against *Streptococcus mutans* biofilms. J. Dent. 170, 106715. doi: 10.1016/j.jdent.2026.106715 42025903

[B113] Wu C. Kim M. J. Mangal U. Seo J. Y. Kim J. Y. Kim J. . (2023). Effect of bacterial resistant zwitterionic derivative incorporation on the physical properties of resin-modified glass ionomer luting cement. Sci. Rep. 13, 3589. doi: 10.1038/s41598-023-30670-4 36869063 PMC9984415

[B99] Wu S. Liu Y. Zhang H. Lei L. (2020). Nano-graphene oxide with antisense vicR RNA reduced exopolysaccharide synthesis and biofilm aggregation for *Streptococcus mutans*. Dent. Mater. J. 39, 278–286. doi: 10.4012/dmj.2019-039 31827056

[B97] Wu R. Zhao Q. Lu S. Fu Y. Yu D. Zhao W. (2018). Inhibitory effect of reduced graphene oxide-silver nanocomposite on progression of artificial enamel caries. J. Appl. Oral. Sci. 27, e20180042. doi: 10.1590/1678-7757-2018-0042 30540069 PMC6296285

[B120] Xu K. Huang R. Li X. Jin L. Ko C. N. Li M. . (2025). Nanomaterial-based synergistic strategies for combating dental caries: Progress and perspectives. Nanoscale 17, 1874–1888. doi: 10.1039/d4nr04515g 39704082

[B135] Ying X. Xue G. Sun P. Gan Z. Fan Z. Liu B. . (2023). Antimicrobial peptides targeting *Streptococcus mutans*: Current research on design, screening and efficacy. Curr. Microbiol. 81, 18. doi: 10.1007/s00284-023-03540-5 38007405

[B25] Yudovin-Farber I. Beyth N. Nyska A. Weiss E. I. Golenser J. Domb A. J. (2008). Surface characterization and biocompatibility of restorative resin containing nanoparticles. Biomacromolecules 9, 3044–3050. doi: 10.1021/bm8004897 18821794

[B26] Yudovin-Farber I. Beyth N. Weiss E. I. Domb A. J. (2010). Antibacterial effect of composite resins containing quaternary ammonium polyethyleneimine nanoparticles. J. Nanopart. Res. 12, 591–603. doi: 10.1007/s11051-009-9628-830311153

[B22] Zaltsman N. Kesler-Shvero D. Weiss E. I. Beyth N. (2016). Synthesis variants of quaternary ammonium polyethyleneimine nanoparticles and their antibacterial efficacy in dental materials. J. Appl. Biomater. Funct. Mater. 14, e205–e211. doi: 10.5301/jabfm.5000269 27032864

[B72] Zane A. Zuo R. Villamena F. A. Rockenbauer A. Digeorge Foushee A. M. Flores K. . (2016). Biocompatibility and antibacterial activity of nitrogen-doped titanium dioxide nanoparticles for use in dental resin formulations. Int. J. Nanomedicine 11, 6459–6470. doi: 10.2147/ijn.s117584 27980404 PMC5147409

[B93] Zanni E. Chandraiahgari C. R. De Bellis G. Montereali M. R. Armiento G. Ballirano P. . (2016). Zinc oxide nanorods-decorated graphene nanoplatelets: a promising antimicrobial agent against the cariogenic bacterium *Streptococcus mutans*. Nanomaterials (Basel) 6, 179. doi: 10.3390/nano6100179 28335307 PMC5245199

[B17] Zhang K. Cheng L. Weir M. D. Bai Y. X. Xu H. H. (2016). Effects of quaternary ammonium chain length on the antibacterial and remineralizing effects of a calcium phosphate nanocomposite. Int. J. Oral. Sci. 8, 45–53. doi: 10.1038/ijos.2015.33 27025265 PMC4822178

[B177] Zhang L. Gao Y. Wang Z. Qi Y. Li L. Wang T. . (2024). Erythrocyte-like mesoporous PDA@CeO2 nanozyme with dual drugs for periodontitis treatment. ACS Appl. Bio Mater. 7, 2851–2861. doi: 10.1021/acsabm.3c01213 38587870

[B109] Zhang N. Ma J. Melo M. A. Weir M. D. Bai Y. Xu H. H. (2015). Protein-repellent and antibacterial dental composite to inhibit biofilms and caries. J. Dent. 43, 225–234. doi: 10.1016/j.jdent.2014.11.008 25478889 PMC4321720

[B108] Zhang N. Melo M. A. Bai Y. Xu H. H. (2014). Novel protein-repellent dental adhesive containing 2-methacryloyloxyethyl phosphorylcholine. J. Dent. 42, 1284–1291. doi: 10.1016/j.jdent.2014.07.016 25234652 PMC4321942

[B71] Zhao Y. Zhang H. Hong L. Zou X. Song J. Han R. . (2023). A multifunctional dental resin composite with Sr-N-doped TiO2 and n-HA fillers for antibacterial and mineralization effects. Int. J. Mol. Sci. 24, 1274. doi: 10.3390/ijms24021274 36674788 PMC9861335

[B175] Zhou Z. Yang Y. Zhou F. Liang K. Gong T. Zhou X. . (2026). Energy-assisted CRISPR cleavage and probiotic vesicle signaling platform: microbiome reprogramming for homeostasis. Sci. Adv. 12, eaef1760. doi: 10.1126/sciadv.aef1760 42497250 PMC13398537

